# Systematic review of central nervous system anomalies in incontinentia pigmenti

**DOI:** 10.1186/1750-1172-8-25

**Published:** 2013-02-13

**Authors:** Snežana Minić, Dušan Trpinac, Miljana Obradović

**Affiliations:** 1 School of Medicine, University of Belgrade, and Dermatovenerology Clinic, Clinical Center of Serbia, Deligradska 34, 11000 Belgrade, Serbia; 2 Institute of Histology and Embryology, School of Medicine, University of Belgrade, Dr Subotića 8, 11000 Belgrade, Serbia; 3 Institute of Histology and Embryology, School of Medicine, University of Belgrade, Dr Subotića 8, 11000 Belgrade, Serbia

**Keywords:** Incontinentia pigmenti, *IKBKG* gene, CNS anomalies, Diagnostic criteria, Systematic review

## Abstract

The objective of this study was to present a systematic review of the central nervous system (CNS) types of anomalies and to consider the possibility to include CNS anomalies in Incontinentia pigmenti (IP) criteria. The analyzed literature data from 1,393 IP cases were from the period 1993–2012. CNS anomalies were diagnosed for 30.44% of the investigated IP patients. The total number of CNS types of anomalies per patient was 1.62. In the present study there was no significantly higher number of anomalies per patient in females than males. The most frequent CNS types of anomalies were seizures, motor impairment, mental retardation, and microcephaly. The most frequently registered CNS lesions found using brain imaging methods were brain infarcts or necrosis, brain atrophies, and corpus callosum lesions. *IKBKG* exon 4–10 deletion was present in 86.00% of genetically confirmed IP patients. The frequency of CNS anomalies, similar to the frequency of retinal anomalies in IP patients, concurrent with their severity, supports their recognition in the list of IP minor criteria.

## Introduction

Incontinentia pigmenti (IP [MIM 308300], Bloch-Sulzberger syndrome, ORPHA464) is a rare X-linked genodermatosis with estimated prevalence 0.2/100,000 [1] in which changes of skin and skin appendages are usually combined with anomalies of other organs: teeth, eyes, and central nervous system (CNS) [2]. IP appears almost exclusively in females and is usually lethal in males [3,4]. Mutations of the *IKBKG* (*inhibitor of kappa B kinase gamma*, previously *NEMO*) gene, localized on the X-chromosome, *locus* Xq28, are responsible for IP [5]. *IKBKG* gene product activates NF-κB (nuclear factor-kappa B), transcription factor that regulates the expression of hundreds of genes in almost all cells [6-9]. The highest expresion level of *IKBKG* was noted in CNS [10]. IP results from enhanced apoptosis due to mutations in the *IKBKG* gene [3]. The phenotypic variability of *IKBKG* mutation expression [5,11] is likely to be the result of skewed X-chromosome inactivation and due to the pleiotropic role of the *IKBKG* gene product [10,12,13]. Proposed criteria for IP were set by Landy and Donnai [3] and have been in routine practice since then. CNS anomalies usually occur from the neonatal through the early infantile period and represent the most important threat to the normal life span of patients with IP [4,14]. CNS anomalies in IP include seizures, mental retardation, hemiplegia, hemiparesis, spasticity, microcephaly, and cerebellar ataxia [3,15].

Since 1906, when IP was described for the first time [16], there have been numerous reports of CNS anomalies in IP patients. We made a systematic review to obtain an overview of relevant aspects of CNS findings in IP. The systematic review covered data in the available literature of IP cases published between 1993, when Landy and Donnai [3] proposed IP criteria, and 2012. The objective of the present study was to determine whether there were any differences between the number and type of anomalies according to sex, to investigate the severity of the anomalies, to discuss possibilities for their correction, and to consider the possibility to include CNS anomalies in IP criteria in the context of their frequency and severity. The discovery of *IKBKG* as the IP causative gene [5] enabled molecular genetic confirmation of IP. The correlation of genetic data and the clinical phenotype, with the main focus on CNS findings, was investigated. Additional data concerning IP literature published before 1993 was also analyzed and presented (see Additional file 1: Central nervous system anomalies in incontinentia pigmenti in the period 1906-1993).

## Methods

Available literature data concerning IP cases published between 1993 and 2012 were analyzed. A literature search was conducted using following databases: PubMed ( http://www.ncbi.nlm.nih.gov/pubmed), National Library of Serbia - KoBSON – Serbian Library Consortium for Coordinated Acquisition of Literature ( http://kobson.nb.rs/kobson.82.html/), Google Scholar ( http://scholar.google.com/schhp?hl=en), and references cited in the articles found. The following search algorithms were used: “Incontinentia pigmenti” and “Bloch-Sulzberger syndrome.” All references with the relevant data independent of article type in English and other languages were included in the study. Clinical data and diagnostic information were collected from abstract or full text of the articles searched for in the databases. Only cases of IP with a confirmed diagnosis, the probands as well as their IP positive relatives ascertained after the diagnosis in the proband, were included. Detailed clinical, brain imaging, and laboratory data needed to be described for case reports. Exact IP diagnostic criteria had to be included for research reports. For systematic review 318 references from the literature with acceptable data for this study were found. Analyzed studies covered the pan-ethnic IP patients’ populations in Europe, Asia, Africa, Australia, North America, and South America. These references are listed separately (see Additional file 2: Additional references published in the period of 1993-2012). The validity of the analyzed articles was taken for granted and was not assessed. Whenever possible we excluded multiplications of data, such as patients described in more than one reference.

In this analysis, the following were considered as CNS anomalies: all types of seizures, motor impairment, mental retardation, microcephaly, and anomalies registered in small number or unspecified anomalies. The following were classified as motor impairment: cerebral palsy, hemiplegia, hemiparesis, spasticity, and ataxia. The number of CNS anomaly types per patient by sex was counted. Quite often, an individual patient had more than one type of CNS anomaly.

Anomalies that produced mild and temporary problems in patients were classified as mild, whereas all anomalies that significantly and permanently affected patients’ health and quality of life were classified as severe.

To collect data about CNS morphological changes in IP patients with neurological symptomatology we analyzed articles in which brain imaging methods, usually MRI (magnetic resonance imaging) and CT (computed tomography), were used. The lesions registered using brain imaging methods were classified as brain infarcts/necrosis, brain atrophies, dilated brain ventricles, brain cystic lesions, corpus callosum lesions (including hypoplasia and agenesis), and rarely diagnosed or unspecified CNS lesions. To determine the effect of different *IKBKG* mutations on the clinical phenotype, the CNS anomalies of IP patients with a proven *IKBKG* mutation were analyzed separately. In addition, the relationship between the occurrence of CNS anomalies and ocular and dental anomalies as the most frequent accompanying disorders in IP patients was investigated. The collected data were mainly frequencies that were presented in tables. All calculations were performed in Microsoft Office Excel™ 2003.

## Results

As outlined above, in the present study the literature data concerning IP patients with associated CNS anomalies were investigated. The results are presented in tables. The total number of IP patients found in the literature during the period 1993–2012 was 1,393 (92.96% females, 6.60% males, and 0.43% without adequate data regarding sex in literature) (Table 1). Among 795 neurologically investigated IP patients, CNS anomalies were observed in 30.44% of them. The total number of CNS types of anomalies per patient was 1.62. The most frequent types of CNS anomalies were seizures, motor impairment, and mental retardation, which comprise 41.98%, 25.70%, and 20.36%, respectively, of all observed types of CNS anomalies. Microcephaly was found in 4.07% of patients, and 7.89% of anomalies were rare or unclassified. Most (61.98%) of IP patients with CNS findings suffered from severe CNS anomalies. The results of the present statistical analysis of data by sex showed that there was no significant difference in the number of types of CNS anomalies per patient (χ^2^=0.42; df=1; *p*=0.71>0.05), in the distribution of patients with mild and severe anomalies (χ^2^=0.00006; p=0.99>0.05), and in the distribution of CNS anomalies (χ^2^=0.21; df=2; *p*=0.90>0.05). The Chi-square test was not used to test distribution by sex of microcephaly and types of CNS anomalies found in small numbers or unspecified because the expected frequency for male IP patients for these CNS anomalies (even when grouped together) was less than five [17].

**Table 1:** The main findings of IP patients and CNS anomalies by sex for the period 1993-2012

	**Number of**					Distribution of CNS anomaly types in numbers **					**Number of**	
**Sex**	**IP patients**	**Neurologically investigated IP patients**	**Neurologically investigated IP patients with CNS anomalies**	**CNS anomalies**	**CNS anomaly types per patient**	**Seizures**	**Mental retardation**	**Motor impairment**	**Microcephaly**	CNS ** anomalies found in small number or unspecified	**Neurologically investigated IP patients with mild CNS anomalies**	**Neurologically investigated IP patients with severe CNS anomalies**
Total*	1,393		795	242	393	1.62	165	80	101	16	31	92	150
**Female**	1,295		719	221	361	1.63	152	73	94	15	27	84	137
**Male**	92		76	21	32	1.52	13	7	7	1	4	8	13

* For six patients, data concerning sex were not available in the literature.

** The task of counting and identifying anomalies in some references was challenging because there were only lists of observed anomalies with no exact numbers. These lists included frequent types of CNS anomalies such as seizures, motor impairment, mental retardation, and microcephaly. These anomalies were classified as unspecified anomalies together with different anomalies presented in small numbers. In this investigation, of 31 registered rare or unspecified CNS anomalies, 15 were unspecified but frequent types of CNS anomalies (seizures, motor impairment, mental retardation, and microcephaly). Because of these difficulties, the exact number of frequent types of CNS anomalies such as seizures, motor impairment, mental retardation, and microcephaly was actually higher than those presented in the tables.

For total of 139 IP patients (6 of them males) we had sufficient data to analyze the age of occurrence of first neurological manifestation. The first neurological manifestations occurred in the first week in 58.3%, in the first month of life in 66.9%, and in the first year in 87.8% IP patients. In all analyzed intervals was registered higher number of severe compared to mild anomalies.

The most frequently registered CNS lesions found using brain imaging methods (Table 2) were brain infarcts or necrosis, brain atrophies, and corpus callosum lesions (24.55%, 17.36%, and 13.17% respectively), whereas rarely diagnosed or unspecified CNS lesions represented 30.54%.

**Table 2:** Distribution of brain imaging lesion findings by sex in IP patients with CNS lesions for the period 1993-2012

**Sex**	**Total number of IP patients with brain imaging lesion findings**	**Total number of brain imaging lesion findings**	**Distribution of brain imaging lesion findings in numbers**					
			**Brain atrophy**	**Brain infarcts/necrosis**	**Dilated brain ventricles**	**Corpus callosum lesions**	**Brain cystic lesions**	**Lesions found in small number or unspecified**
**Total**	89	167	29	41	11	22	13	51
**Female**	84	161	27	39	10	22	13	50
**Male**	5	6	2	2	1	0	0	1

*IKBKG* mutations were confirmed for 50 IP patients with CNS anomalies (Table 3). They represent 11.11% of the total number of registered IP patients with CNS anomalies. Three of them were males, and all had *IKBKG* exon 4–10 deletion. In IP patients positive for *IKBKG* mutations (Table 4) 86.00% were found to have *IKBKG* exon 4–10 deletion whereas 14.00% had other than *IKBKG* exon 4–10 mutation listed in Table 5. The total number of CNS types of anomalies per patient was 1.54 in IP patients with genetically confirmed IP and 1.62 in patients without genetically confirmed IP. The total number of CNS anomalies per patient was 1.42 in IP patients with a common exon 4–10 deletion and 2.28 in IP patients with other types of *IKBKG* mutations. The result of the present statistical analysis according to genetical confirmation showed that there was no significant difference in the number of CNS anomaly types per patient (χ^2^=0.17; df=1; *p*=0.68>0.05) or in the distribution of seizures, mental retardation, and motor impairment (χ^2^=2.16; df=2; *p*=0.54>0.05). Severe CNS types of anomalies were found in 63.22% of IP patients with CNS anomalies without genetically confirmed IP and in 54.00% of IP patients with CNS types of anomalies that were positively genetically tested for the *IKBKG* mutation, but the difference was not significant (χ^2^=1.35; df=1; *p*=0.24>0.05).

**Table 3:** **The main findings of genetically analyzed IP patients and CNS anomalies according to the presence of genetically confirmed *IKBKG *mutation for the period 2001-2012**

**IP patients with and without genetical confirmation of the *IKBKG *mutation**	**Number of**			**Distribution of CNS anomaly types in numbers**					**Distribution of IP patients according to severity of anomalies in numbers**	
	**Neurologically investigated IP patients with CNS anomalies**	**CNS anomaly types**	**CNS anomaly types per patient**	**Seizures**	**Mental retardation**	**Motor impairment**	**Microcephaly**	**CNS anomalies found in small number or unspecified**	**IP patients with mild CNS anomalies**	**IP patients with severe CNS anomalies**
**Total**	205		329	1.60	141	66	82	12	28	80	125
**IP patients without genetical confirmation**	155		252	1.62	111	55	69	9	8	57	98
**IP patients with genetical confirmation**	50		77	1.54	30	11	13	3	20	23	27

**Table 4:** **Number of IP patients with CNS anomalies and confirmed *IKBKG *mutations, CNS anomaly types, and its distribution according to type of *IKBKG *mutation for the 2001–2012 period**

**Type of *IKBKG *mutation**	**Total number of**				**Distribution of CNS types of anomalies in number**				
	**Genetically confirmed IP patients with CNS anomalies**	**CNS anomaly types in genetically confirmed IP patients**	**CNS anomaly types per patient**	**Genetically confirmed IP patients with confirmed imaging lesion findings**	**Seizures**	**Mental retardation**	**Motor impairment**	**Microcephaly**	**CNS anomaly types found in small number, unspecified or asymptomatic**
**All *IKBKG *mutations**	50*		77	1.54	28	30	11	13	3	20
***IKBKG *exon 4–10 deletion**	43*		61	1.42	27	28	9	9	1	14
**Other than *IKBKG *exon 4–10 deletion**	7**		16	2.28	1	2	2	4	2	6

* Three of them were males.

** All 7 patients were females.

**Table 5:** **List of *IKBKG *mutations except *IKBKG *exon 4–10 deletion for the 2001–2012 period in IP patients with CNS anomalies**

**Reference**	**Type of *IKBKG *mutation**	**Type of anomalies**	**Diagnosis**
Rola et al. 2004 [18]	Nonsense mutation c.397C>T (p.Gln133Term)	Motor impairment	Incontinentia pigmenti
Fusco et al. 2004 [12]	266–269delAGA	Seizures	Incontinentia pigmenti
		Spastic paresis	
		Mental/motor retardation	
		Microcephaly	
	1150C!T	1 unspecified* frequent anomaly	Incontinentia pigmenti
	1077–1078delC	3 unspecified* frequent anomalies	Incontinentia pigmenti
	1115–1116delT	1 unspecified* frequent anomaly	Incontinentia pigmenti
Martinez-Pomar et al. 2005 [19]	Frameshift mutation c.792dupA (p.Gln265ThrfsX19)	Motor impairment	Incontinentia pigmenti, transient immunodeficiency
Sebban-Benin et al. 2007 [11]	Missense mutation c.967G>C (p.Ala323Pro)	Seizures	Incontinentia pigmenti
		Mental retardation	
		Motor impairment	
		Microcephaly	

All IP patients were females.

* Unspecified frequent anomalies: seizures, motor impairment, mental retardation and microcephaly.

In this analysis of literature data, of 242 IP patients with CNS anomalies, 186 were ophthalmologically investigated, and 101 (54.30%) of them had associated ocular anomalies. A total of 115 IP patients were investigated stomatologically, and 80 (69.56%) of them had dental and/or oral anomalies. A total of 41 (36.93%) IP patients had CNS, ocular, and dental and/or oral anomalies simultaneously.

## Discussion

The results of this investigation presented the distribution by sex similar to the results of other studies [2,15]. The percentage of IP patients with CNS anomalies was similar to the results of Carney [15] and Hadj-Rabia et al. [20], 30.5% and 32%, respectively. However, Fusco et al. [12] identified 13% and Kim et al. [21] identified 35% IP patients with CNS anomalies. These discrepancies could be explained by the differences in the IP patients’ cohort. The results of present statistical analysis of data by sex showed that there was no significant difference in the number of CNS anomaly types per patient or in the distribution of seizures, mental retardation, and motor impairment.

Besides limitations such as heterogeneity, different definitions, and criteria for their diagnosis, frequencies of seizures, motor impairment, mental retardation, and microcephaly were generally higher than in the corresponding general population. For example, according to Eurocat Prevalence Data Tables [22] microcephaly for live births for the 2005–2009 period was 1.67 per 10,000 births. In the general population the prevalence of epilepsy was 0.005-0.01% [23], and the prevalence of mental retardation was 1-3% [24].

In the present study there was no significantly higher number of anomalies per patient in females than males. Fusco et al. [12,25] found higher values, 2.25 CNS anomaly types per patient in females and 2.40 in males, whereas Kim et al. [21] found only one CNS anomaly type per patient. Fusco et al. [12,25] found 13.55% in female and 35.71% in male IP patients with CNS anomalies. These differences in findings very likely originate from different sample sizes, and, because of the larger sample size, the results of the present study were more reliable.

Because pathoanatomical findings in IP patients were very rare [26-28], brain imaging methods gave the majority of data concerning the morphological aspects of CNS lesions [29]. The most frequently registered CNS lesions with the brain imaging method were brain infarcts or necrosis, brain atrophy, and corpus callosum lesions.

Besides the fact that IP results from enhanced apoptosis due to mutations in the *IKBKG* gene [3,4,10], pathogenesis of CNS lesions in IP is still controversial issue. The CNS lesions may share a common pathophysiologic state with the vascular occlusive disease seen in the retinas of IP patients [30-32]. However, other brain imaging and pathoanatomical studies fail to show a relationship between brain abnormalities and vascular patterns [14,26]. Brain infarcts found in IP patients [30-32] supported the hypothesis of vascular pathogenesis of CNS lesions in IP, whereas findings of brain atrophy [11,20], corpus callosum lesions [14,20], disorder of myelination [27,33] and lack of relationship between brain abnormalities and vascular patterns [14,26] supported the hypothesis of disorder of the NF-κB metabolic pathway in neurons and glia cells as a pathogenetic mechanism.

CNS lesions in IP can result from the same pathogenesis as skin [4], by inducing apoptosis of *IKBKG* mutated cells because the CNS, such as the skin, is of ectodermal origin. The evolution of the lesions can be interpreted as representing the death of cells that have the *IKBKG* mutation-bearing X-chromosome as the active one and their replacement by cells in which the normal X-chromosome is active [34]. Considering the similarities between IP retinopathy and retinopathies of prematurity [10,35] and in diabetes [35], the fact that apoptosis in the diabetic rat retina occurred before changes in microcirculation [35] indicates that a similar process may occur in the retina and the CNS of IP patients. One must bear in mind the fact that apoptosis is a process defined by manifesting specific morphological and molecular features, visible only in histological sections under microscope [36,37], not visible directly and clinically in patient. It is possible that in the CNS, as in the retina, apoptotic changes precede vascular changes, but the first clinically noticeable signs in IP patients are vascular changes. In the experimental model of retinopathy of prematurity occurred apoptosis of endothelial cells in the developing retina, leading to vaso-obliteration followed by proliferative retinopathy [38]. It is possible to speculate that, besides parenchymal cells, apoptosis occurs in blood vessel walls’ cells of affected organs. For the time being there is no evidence for such a hypothesis.

Besides apoptosis, some other processes occurred in *IKBKG* mutation affected tissue. *IKBKG* mutation leads to expression of eotaxin, chemokine chemotactic for eosinophils [39] responsible for eosinophil infiltration [40]. Eotaxin is abnormally expressed in epidermis affected by *IKBKG* mutation and in vascular endothelial cells, findings which correlate with eosinophil infiltration in the skin [40]. Perivascular eosinophil deposition has been observed along with endothelial cell degeneration within retinal blood vessels [41]. It was proposed that apoptotic keratinocytes act leading to inflammatory responses inducing synthesis and release of different chemokines including eotaxin and sequent eosinophil infiltration [40]. There is also proposal that vascular endothelial growth factor (VEGF) receptor is involved in the vascular changes in IP [10,26]. As NF-κB is involved in the transduction system of the messages received by the VEGF receptor, disorder of the transmission of this message in IP might influence cerebral microvascularization [10,26].

Taking into account all the facts presented, identical pathophysiological mechanisms of IP development with apoptosis as a key event occur in all affected tissues, skin, retina, and CNS while the vascular changes in IP appear secondary to apoptosis.

A group of 50 IP patients from the literature with CNS anomalies that were positive tested for *IKBKG* mutations was independently analyzed. The percentage of IP patients with common exon 4–10 deletion is similar to the results (80%) of Smahi and The International Incontinentia Pigmenti Consortium [5]. Although the number of CNS anomaly types was higher for common *IKBKG* exon 4–10 deletion, the difference was not significant. Fusco et al. [25] found a similar distribution: 2.00 CNS anomaly types per IP patient with *IKBKG* exon 4–10 deletion and 2.50 CNS anomaly types per IP patient with other than *IKBKG* exon 4–10 deletion. However, because of the low number of documented patients with other than *IKBKG* exon 4–10 mutations this can be also the result of a statistical bias. The correlation between type of *IKBKG* mutation and IP phenotype expression was not found up to date.

We investigated the relationship of the simultaneous occurrence of the most frequent IP extracutaneous anomalies: CNS, ocular, and dental and/or oral anomalies. Based on literature data of a selected series of 29 IP patients with MRI confirmed CNS anomalies, from which 20 (68.96%) had associated ocular anomalies, it was speculated that ocular anomalies increase the chance of CNS anomalies in IP patients [30]. However, Fusco et al. [12] in a series of 59 genetically confirmed IP females found eight patients with CNS anomalies, only four of whom had associated ocular anomalies. In the Hadj-Rabia et al. [20] study only three of 13 IP patients with CNS anomalies had associated ocular anomalies. In the present analyses of literature data 101 (54.30%) of the IP patients had associated ocular anomalies. In extensive meta-analyses 37.44% (289/772) of IP patients with diagnosed eye anomalies were found [42]. The assumption that ocular anomalies increase the chance of CNS anomalies in IP patients [30] was made based on a very specific and relatively small sample. The high number of accompanying ocular anomalies reflected primarily common embryonic origin of eyes and CNS. Besides ocular anomalies, dental and/or oral anomalies were analyzed. They were present in 80 (69.56%) IP patients with CNS anomalies. There was a higher correlation between CNS and dental and/or oral anomalies than CNS and ocular anomalies. In an extensive systematic review dental and/or oral anomalies were diagnosed for 54.38% (279/513) of the investigated IP patients [43]. A total of 41 IP patients had CNS, ocular, and dental and/or oral anomalies simultaneously. Different combinations of associated extracutaneous anomalies in IP are likely to be the result of skewed X-chromosome inactivation and due to the pleiotropic role of *IKBKG* gene product [10,12,44].

Because the epidermis, a key substrate of IP, and CNS had same embryonic origin, it was supposed that IP CNS changes are in accordance with their origin [4]. However, besides the fact that *IKBKG* gene mutations were considered as the only cause of IP phenotype [4], one must consider that other possibilities for their origin may exist. This problem is related not only to CNS anomalies associated with IP. A similar situation with dental and oral anomalies was recently discussed in detail [43]. Because *IKBKG* is involved in a complex NF-κB signaling pathway that regulates the expression of hundreds of genes [6], its mutation can produce different disorders in organisms, and the entire spectrum of anomalies seen in IP usually is attributed to *IKBKG* mutations. Statistical data indicate that incidences of typical CNS anomalies in IP were much higher than in the general population. However, there are no absolute facts that exclude the possibility that some other gene mutations in IP patients besides *IKBKG* mutations exist [44]. There are a great number of genes whose mutations are responsible for different anomalies, including some CNS anomalies found in IP: mental retardation, seizure, and microcephaly. Besides involvement of *IKBKG* gene mutation in mental retardation that is generally recognized [45], there are more than 290 other genes involved in mental retardation [46]. Thus, it is hypothetically possible that cutaneous manifestations in IP originate from *IKBKG* mutation, whereas extracutaneous anomalies in IP patients originate from some gene mutation other than *IKBKG*. According to this hypothesis there is an option that combinations of mutations of *IKBKG* and some other gene(s) are responsible for final IP phenotype expression – skin changes and associated extracutaneous anomalies. In the available literature there are no facts to confirm or reject such a hypothesis. So the possibility of the existence of a few different mutations (including *IKBKG*) responsible for the complete phenotypic characteristics of IP is still open.

According to Landy and Donnai’s IP diagnostic criteria [3], skin lesions were classified as IP major criteria, whereas dental, hair, nail, and retinal anomalies were classified as IP minor criteria. Landy and Donnai [3] registered a high percentage of CNS anomalies (<10%) in their unpublished series of 111 IP patients, but omitted CNS anomalies as IP minor criteria. Histological features of affected skin and nipple anomalies as well as oral anomalies, especially palate anomalies, were also omitted so far as minor criteria [43,47]. To establish the possibility to include CNS anomalies as IP minor criteria, percentages of severe CNS anomalies and retinal anomalies in IP were compared. In our meta-analysis of eye anomalies in IP patients for the 1976–2010 period, 37.44% of IP patients with diagnosed eye anomalies were found [42]. Retinal anomalies were present in 17.52% of IP patients [42]. Based on collected and analyzed data it is obvious that the total percentage of IP patients with CNS (30.44%) and ocular anomalies (37.44%) [42] is similar, as is the percentage of IP patients with vision-threatening retinal (17.52%) [42] and severe CNS (18.86%) anomalies. Taking into account that vision-threatening retinal anomalies, already recognized as IP minor criteria [3], were registered in a smaller percentage than severe CNS anomalies and the fact that CNS anomalies represent the most important threat to the normal life span of patients with IP [4], CNS anomalies should be included as IP minor criteria.

Currently causative therapy for IP, including associated CNS anomalies, does not exist. CNS anomalies are life threatening and are the most severe consequences of IP. Because CNS anomalies usually occur from the neonatal through the early infantile period, fast referral of neonates with IP to pediatric neurologists should be routinely considered.

## Conclusion

CNS anomalies in IP patients pose a serious threat to the normal life of an IP patient. The frequency of CNS anomalies, similar to the frequency of retinal anomalies in IP patients, concurrent with their severity, supports their recognition in the list of IP minor criteria.

## Abbreviations

IP: Incontinentia pigmenti; *IKBKG*: Inhibitor of kappa B kinase gamma; *NEMO*: NF-κB essential modulator; NF-κB: Nuclear factor-kappa B; CNS: Central nervous system; MRI: Magnetic resonance imaging; CT: Computed tomography; VEGF: Vascular endothelial growth factor.

## Competing interests

The authors declare that they have no competing interests.

## Authors’ contributions

SM carried out basic conception and design of study, analysis and interpretation of data, were involved in writing the manuscript and revising it critically for important intellectual content. DT contributed in establishing conception and design of study, acquisition and analysis of data, statistical analysis and was involved in writing and revising the manuscript. MO participated in acquisition and interpretation of data; helped to draft the manuscript and participated in revising it critically. All authors read and approved the final manuscript.

## Supplementary Material

Additional file 1 Central nervous system anomalies in incontinentia pigmenti in the perod 1906-1993. Click here for file

Additional file 2 Additional references published in the period of 1993-2012. Click here for file

## References

[B1] Orphanet Report Series - Prevalence of rare diseases: Bibliographic data - May 2012 – Number 1 [ http://www.orpha.net/orphacom/cahiers/docs/GB/Prevalence_of_rare_diseases_by_alphabetical_list.pdf]

[B2] Scheuerle A Ursini MV Incontinentia pigmenti (Bloch-Sulzberger syndrome) University of Washington, Seattle (WA): GeneReviews 2008 Feb, 22 pp. [ http://www.ncbi.nlm.nih.gov/bookshelf/br.fcgi?book=gene&part=i-p.]

[B3] Landy SJ Donnai D Incontinentia pigmenti (Bloch-Sulzberger syndrome) J Med Genet 1993 30 53 59 10.1136/jmg.30.1.53 8423608 PMC1016235

[B4] Berlin AL Paller AS Chan LS Incontinentia pigmenti: a review and update on the molecular basis of pathophysiology J Am Acad Dermatol 2002 47 169 187 10.1067/mjd.2002.125949 12140463

[B5] Smahi A Courtois G Vabres P Yamaoka S Heuertz S Munnich A Israël A Heiss NS Klauck SM Kioschis P Wiemann S Poustka A Esposito T Bardaro T Gianfrancesco F Ciccodicola A D'Urso M Woffendin H Jakins T Donnai D Stewart H Kenwrick SJ Aradhya S Yamagata T Levy M Lewis RA Nelson DL Genomic rearrangement in NEMO impairs NF-κB activation and is a cause of incontinentia pigmenti. The International Incontinentia Pigmenti (IP) Consortium Nature 2000 405 466 472 10.1038/35013114 10839543

[B6] Courtois G Gilmore TD Mutations in the NF-κB signaling pathway: implication for human disease Oncogene 2006 25 6831 6843 10.1038/sj.onc.1209939 17072331

[B7] Mattson MP Camandola S NF-κB in neuronal plasticity and neurodegenerative disorders J Clin Invest 2001 107 247 254 10.1172/JCI11916 11160145 PMC199201

[B8] Mattson MP Meffert MK Roles for NF-κB in nerve cell survival, plasticity, and disease Cell Death Differ 2006 13 852 860 10.1038/sj.cdd.4401837 16397579

[B9] Levenson JM Pizzi M Sweatt JD Liou H-C NF-κB in neurons: behavioral and physiologic roles in nervous system function NF-kB/Rel transcription factor family 2006 Georgtown and New York: Landes Bioscience and Springer Science+Business Media 147 161

[B10] Aradhya S Woffendin H Jakins T Bardaro T Esposito T Smahi A Shaw C Levy M Munnich A D'Urso M Lewis RA Kenwrick S Nelson DL A recurrent deletion in the ubiquitously expressed NEMO (IKK-γ) gene accounts for the vast majority of incontinentia pigmenti mutations Hum Mol Genet 2001 10 2171 2179 10.1093/hmg/10.19.2171 11590134

[B11] Sebban-Benin H Pescatore A Fusco F Pascuale V Gautheron J Yamaoka S Moncla A Ursini MV Identification of TRAF6-dependent NEMO polyubiquitination sites through analysis of a new NEMO mutation causing incontinentia pigmenti Hum Mol Genet 2007 16 2805 2815 10.1093/hmg/ddm237 17728323

[B12] Fusco F Bardaro T Fimiani G Mercadante V Miano MG Falco G Israël A Courtois G D'Urso M Ursini MV Molecular analysis of the genetic defect in a large cohort of IP patients and identification of novel NEMO mutations interfering with NF-κB activation Hum Mol Genet 2004 13 1763 1773 10.1093/hmg/ddh192 15229184

[B13] Fusco F Pescatore A Bal E Ghoul A Paciolla M Lioi MB D'Urso M Rabia SH Bodemer C Bonnefont JP Munnich A Miano MG Smahi A Ursini MV Alterations of the IKBKG locus and diseases: an update and a report of 13 novel mutations Hum Mutat 2008 29 595 604 10.1002/humu.20739 18350553

[B14] Pascual-Castroviejo I Pascual-Pascual SI Velázquez-Fragua R Martinez V Incontinentia pigmenti: clinical and neuroimaging findings in a series of 12 patients Neurologia 2006 21 239 248 16788866

[B15] Carney RG Incontinentia pigmenti: a world statistical analysis Arch Dermatol 1976 112 535 542 10.1001/archderm.1976.01630280059017 1267462

[B16] Garrod AE Peculiar pigmentation of the skin of an infant Trans Clin Soc London 1906 39 216

[B17] Sachs L Applied statistics 1984 2 New York: Springer-Verlag

[B18] Rola M Martins T Melo MJ Gomes R Roseira J Souto A Incontinence of pigment An Pediatr (Barc) 2004 60 601 602 10.1157/13062338 15207182

[B19] Martinez-Pomar N Munoz-Saa I Heine-Suner A Martin A Smahi A Metamoros N A new mutation in exon 7 of NEMO gene: late skewed X-chromosome inactivation in an incontinentia pigmenti female patient with immunodeficiency Hum Genet 2005 118 458 465 10.1007/s00439-005-0068-y 16228229

[B20] Hadj-Rabia S Froidevaux D Bodak N Hamel-Teillac D Smahi A Touil Y Fraitag S de Prost Y Bodemer C Clinical study of 40 cases of incontinentia pigmenti Arch Dermatol 2003 139 1163 1170 10.1001/archderm.139.9.1163 12975158

[B21] Kim BJ Shin HS Won CH Lee JH Kim KH Kim MN Ro BI Kwon OS Incontinentia pigmenti: clinical observation of 40 Korean cases J Korean Med Sci 2006 21 474 477 10.3346/jkms.2006.21.3.474 16778392 PMC2729954

[B22] Eurocat Prevalence Data Tables [ http://www.eurocat-network.eu/prevdata/resultsPdf.aspx?title=B3&allanom=false&allregf=true&allrega=true&anomalies=8&winx=1256&winy=894]

[B23] Theodore WH Spencer SS Wiebe S Langfitt JT Ali A Shafer PP Berg AT Vickrey BG Epilepsy in North America: a report prepared under the auspices of the global campaign against epilepsy, the International Bureau for Epilepsy, the International League Against Epilepsy, and the World Health Organization Epilepsia 2006 47 1700 1722 10.1111/j.1528-1167.2006.00633.x 17054693

[B24] Galasso C Lo-Castro A El-Malhany N Curatolo P "Idiopathic" mental retardation and new chromosomal abnormalities Ital J Pediatr 2010 36 17 10.1186/1824-7288-36-17 20152051 PMC2844383

[B25] Fusco F Fimiani G Tadini G Michele D Ursini MV Clinical diagnosis of incontinentia pigmenti in a cohort of male patients J Am Acad Dermatol 2007 56 264 267 10.1016/j.jaad.2006.09.019 17224368

[B26] Bachevalier F Marchal C Di Cesare MP Antunes A Truchetet F Atteinte neurologique létale au cours d'une incontinentia pigmenti Ann Dermatol Venereol 2003 130 1139 1142 AD-12-2003-130-12-0151-9638-101019-ART8 14724517

[B27] Hauw JJ Perié G Bonnette J Escourolle R Les 1ésions cérébrales de l’incontinentia pigmenti. A propos d’un cas anatomique Acta Neuropathol 1977 38 159 162 10.1007/BF00688564 878852

[B28] Buinauskiene J Buinauskaite E Valiukeviciene S Incontinentia pigmenti (Bloch-Sulzberger syndrome) in neonates Medicina (Kaunas) 2005 41 496 499 15998988

[B29] Edelstein S Naidich TP Newton TH Tortori-Donati P, Rossi A The rare phakomatoses Pediatric neuroradiology. Part I 2005 Berlin-Heidelberg: Springer-Verlag 819 854

[B30] Meuwissen MEC Mancini GMS Neurological findings in incontinentia pigmenti; a review Eur J Med Genet 2012 55 323 331 10.1016/j.ejmg.2012.04.007 22564885

[B31] Fiorillo L Sinclair DB O'Byrne ML Krol AL Bilateral cerebrovascular accidents in incontinentia pigmenti Pediatr Neurol 2003 29 66 68 10.1016/S0887-8994(03)00144-9 13679126

[B32] Hennel SJ Ekert PG Volpe JJ Inder TE Insights into the pathogenesis of cerebral lesions in incontinentia pigmenti Pediatr Neurol 2003 29 148 150 10.1016/S0887-8994(03)00150-4 14580659

[B33] Philippe O Rio M Malan V Van Esch H Baujat G Bahi-Buisson N Valayannopoulos V Gesny R Bonnefont JP Munnich A Froyen G Amiel J Boddaert N Colleaux L NF-κB signalling requirement for brain myelin formation is shown by genotype/MRI phenotype correlations in patients with Xq28 duplications Eur J Hum Genet 2012 10.1038/ejhg.2012.140 PMC3548256 22805531

[B34] Van der Knaap M Valk J Magnetic resonance of myelination and myelin disorders 2005 Berlin-Heidelberg: Springer

[B35] Hu YB Zhang JK Sun ZY Yuan ZG Liu YM Mao CJ Yan H Retinal cell apoptosis, microvascular changes and expression of connective tissue growth factor in experimental diabetic rats Zhonghua Yan Ke Za Zhi 2011 47 521 526 21914267

[B36] Kroemer G Galluzzi L Vandenabeele P Abrams J Alnemri ES Baehrecke EH Blagosklonny MV El-Deiry WS Golstein P Green DR Hengartner M Knight RA Kumar S Lipton SA Malorni W Nuñez G Peter ME Tschopp J Yuan J Piacentini M Zhivotovsky B Melino G Nomenclature Committee on Cell Death 2009 Classification of cell death: recommendations of the Nomenclature Committee on Cell Death 2009 Cell Death Differ 2009 16 3 11 10.1038/cdd.2008.150 18846107 PMC2744427

[B37] Galluzzi L Vitale I Abrams JM Alnemri ES Baehrecke EH Blagosklonny MV Dawson TM Dawson VL El-Deiry WS Fulda S Gottlieb E Green DR Hengartner MO Kepp O Knight RA Kumar S Lipton SA Lu X Madeo F Malorni W Mehlen P Nuñez G Peter ME Piacentini M Rubinsztein DC Shi Y Simon HU Vandenabeele P White E Yuan J Molecular definitions of cell death subroutines: recommendations of the Nomenclature Committee on Cell Death 2012 Cell Death Differ 2012 19 107 120 10.1038/cdd.2011.96 21760595 PMC3252826

[B38] Gu X El-Remessy AB Brooks SE Al-Shabrawey M Tsai NT Caldwell RB Hyperoxia induces retinal vascular endothelial cell apoptosis through formation of peroxynitrite Am J Physiol Cell Physiol 2003 285 C546 C554 10.1152/ajpcell.00424.2002 12736139

[B39] Garcia-Zepeda EA Rothenberg ME Ownbey RT Celstin J Leder P Luster AD Human eotaxin is a specific chemoattractant for eosinophil cells and provides a new mechanism to explain tissue eosinophilia Nat Med 1996 2 449 456 10.1038/nm0496-449 8597956

[B40] Jean-Baptiste S O’Toole EA Chen M Guitart J Paller A Chan LS Expression of eotaxin, an eosinophil-selective chemokine, parallels eosinophil accumulation in the vesiculobullous stage of incontinentia pigmenti Clin Exp Immunol 2002 127 470 478 10.1046/j.1365-2249.2002.01755.x 11966763 PMC1906303

[B41] Goldberg MF The skin is not the predominant problem in incontinentia pigmenti Arch Dermatol 2004 140 748 750 10.1001/archderm.140.6.748 15210470

[B42] Minić S Obradović M Kovačević I Trpinac D Ocular anomalies in incontinentia pigmenti - literature review and meta-analysis Srp Arh Celok Lek 2010 138 408 413 10.2298/SARH1008408M 20842883

[B43] Minić S Trpinac D Gabriel H Gencik M Obradović M Dental and oral anomalies in incontinentia pigmenti: a systematic review Clin Oral Invest 2013 17 1 8 10.1007/s00784-012-0721-5 22453515

[B44] Fusco F Paciolla M Napolitano F Pescatore A D'Addario I Bal E Lioi MB Smahi A Miano MG Ursini MV Genomic architecture at the Incontinentia Pigmenti locus favours de novo pathological alleles through different mechanisms Hum Mol Genet 2012 21 1260 1271 10.1093/hmg/ddr556 22121116

[B45] Chiurazzi P Schwartz CE Gecz J Neri G XLMR genes: update 2007 Eur J Hum Genet 2008 16 422 434 10.1038/sj.ejhg.5201994 18197188

[B46] Chelly J Khelfaoui M Francis F Chérif B Bienvenu T Genetics and pathophysiology of mental retardation Eur J Hum Genet 2006 14 701 713 10.1038/sj.ejhg.5201595 16721406

[B47] Hadj-Rabia S Rimella A Smahi A Fraitag S Hamel-Teillac D Bonnefont JP de Prost Y Bodemer C Clinical and histologic features of incontinentia pigmenti in adults with nuclear factor-κB essential modulator gene mutations J Am Acad Dermatol 2011 64 508 515 10.1016/j.jaad.2010.01.045 21255870

