# Supplementary material for: Systematic review of central nervous system anomalies in incontinentia pigmenti
Source: Orphanet J Rare Dis. 2013 Feb 13;8:25. doi: 10.1186/1750-1172-8-25 (PMC3576363; doi:10.1186/1750-1172-8-25)
Supplement: Additional file 1 — Central nervous system anomalies in incontinentia pigmenti in the perod 1906-1993. [file 1750-1172-8-25-S1.pdf]

Systematic review of central nervous system anomalies in incontinentia pigmenti

Snežana Minić, Dušan Trpinac, Miljana Obradović

S. Minić
School of Medicine, University of Belgrade, and
Dermatovenerology Clinics, Clinical Center of Serbia,
Deligradska 34, 11000 Belgrade, Serbia
e-mail: dtrpinac@eunet.rs
Tel.: +381-64-199-88-67
Fax: +381-11-361-25-67

Additional file 1

Central nervous system anomalies in incontinentia pigmenti in the perod 1906-1993

In addition to investigation of the period 1993-2012, 82 references for the periods 1906-1975 and 1976-1992 were collected in the same manner and were analyzed, comparing results from three different periods. The data of IP patients for the period between 1906 and 1975 were used from Carney's review [1976]. These references are listed in Additional references published in the period of 1906-1993.

The results of the present systematic review for the period 1906-2012 are presented in Additional Table 1. It contains the summarized data of IP patients, CNS anomalies, and their distribution by sex and by the period of investigation. The data of IP patients for the period 1906-1975 were from Carney's review [1976], where the data for the periods 1976-1992 and 1993-2012 refers to IP patients for which data from the literature were collected.

The total number of IP patients found in the literature for the 1906-2012 period was 2,291 (91.83% females, 5.85% males, and 2.32% with unspecified sex). There were 752 CNS anomalies registered in 450 IP patients for the period 1906-2012. The total number of CNS anomaly types per patient for the periods 1906-1975, 1976-1992, and 1993-2012 were 1.81, 1.54, and 1.62, respectively. For the three investigated periods, no significant difference in the total of CNS anomaly types per patient (χ^2=2.58; df=2; p=0.28>0.05) was found. Among neurologically investigated IP patients, CNS anomalies were observed in 31.25% of the patients in the cumulative period 1906-2012. The proportions of patients with CNS anomalies for the periods 1906-1975, 1976-1992, and 1993-2012 were 30.54%, 36.67%, and 30.44%, respectively, and they did not differ significantly (χ^2=1.93; df=2; p=0.38>0.05). Only for the 1976-1992 and 1993-2012 periods were data adequate for calculating the number of CNS anomalies according to sex. For the periods 1976-1992 and 1993-2012 higher values for the percentage of female IP patients with CNS anomalies than in males, 38.55% and 15.38% (1976-1992) and 30.74% and 27.63% (1993-2012), respectively, were found. In the present study for the periods 1976-1992 and 1993-2012 there was no significantly higher number of anomalies per patient in females than males, 1.55 and 1.50 (1976-1992) and 1.63 and 1.52 (1993-2012), respectively. For the cumulative 1906-2012 period the most frequent CNS anomalies were seizures, motor impairment, and mental retardation, which comprise 34.04%, 27.66%, and 24.73% of the total number of anomalies, respectively. Microcephaly was found in 5.58%, and unspecified anomalies were found in 7.98%. For the three investigated periods a significant difference in the distribution of CNS anomaly types (χ^2=27.47; df=8; p=0.001<0.05) was found. The difference is also significant for three anomaly types: seizures, mental retardation, and motor impairment (χ^2=22.95; df=4; p=0.0001<0.05). For the three investigated periods a significant difference in the distribution of patients with mild and severe CNS anomalies (χ^2=15.36; df=2; p=0.001<0.05) was also found.

Additional Table 1 Main findings of IP patients and CNS anomalies by sex and for different periods of investigation

Observed period and sex	Number of					Distribution of CNS anomaly types in numbers					Percentage of		
	IP patients	Neurologically investigated IP patients	Neurologically investigated IP patients with CNS anomalies	CNS anomalies	CNS anomaly types per patient	Seizures	Mental retardation	Motor impairment	Microcephaly	CNS anomalies found in small number or unspecified	Neurologically investigated IP patients with CNS anomalies	IP patients with mild CNS anomalies	IP patients with severe CNS anomalies
Total* 1906-2012	2,291	1440	450	752	1.67	256	186	208	42	60***	31.25	11.80	19.45
Female 1906-2012	2,104	1339	422	712	1.69	242	177	200	41	52	31.52	11.79	19.72
Male 1906-2012	134	100	28	40	1.43	14	9	8	1	8	28.00	13.00	15.00
Total* 1906-1975	653	465	142	257	1.81	62	76	77	19	23	30.54	14.19	16.34
Female 1906-1975	593	454	137	252	1.84	62	75	77	19	19	30.18	13.66	16.52
Male 1906-1975	16	11	5	5**	1**	0	1	0	0	4	45.46	36.36	9.09
Total* 1976-1992	245	180	66	102	1.54	29	30	30	7	6	36.67	6.67	30.00
Female 1976-1992	216	166	64	99	1.55	28	29	29	7	6	38.55	6.62	31.93
Male 1976-1992	26	13	2	3	1.50	1	1	1	0	0	15.38	7.69	7.69
Total* 1993-2012	1,393	795	242	393	1.62	165	80	101	16	31***	30.44	11.57	18.86
Female 1993-2012	1,295	719	221	361	1.63	152	73	94	15	27	30.74	11.68	19.05
Male 1993-2012	92	76	21	32	1.52	13	7	7	1	4	27.63	10.52	17.10

*In all observed periods there were some patients without adequate data regarding sex in literature so total number of patients is always higher then sum of females and males.

**Insufficient data for exact calculating because adequate detailed information about number and type of CNS anomalies was not available.

***The task of counting and identifying anomalies in some references was challenging because there were only lists of observed anomalies with no exact numbers. These lists included frequent types of CNS anomalies such as seizures, motor impairment, mental retardation, and microcephaly. These anomalies were classified as unspecified anomalies together with different anomalies presented in small numbers. In this investigation, for the period 1993-2012, of 31 registered rare or unspecified CNS anomalies, 15 were unspecified but frequent types of CNS anomalies (seizures, motor impairment, mental retardation, and microcephaly). Because of these difficulties, the exact number of frequent types of CNS anomalies such as seizures, motor impairment, mental retardation, and microcephaly was actually higher than those presented in the Table.

Additional references published in the period of 1906-1992

1. Ashley JR, Burgdorf WH: **Incontinentia pigmenti: pigmentary changes independent of incontinence.** *J Cutan Pathol* 1987, **14**:248–250.
2. Attramadal A: **Incontinentia pigmenti.** *Nor Tannlaegeforen Tid* 1978, **88**:143–146.
3. Avrahami E, Harel S, Jurgenson U, Cohn DF: **Computed tomographic demonstration of brain changes in incontinentia pigmenti.** *Am J Dis Child* 1985, **139**:372–374.
4. Barson WJ, Reiner CB: **Coxsackievirus B2 infection in a neonate with incontinentia pigmenti.** *Pediatrics* 1986, **77**:897–900.
5. Bleehen SS, Wood ML: **Incontinentia pigmenti. Bloch-Sulzberger syndrome.** *Br J Dermatol* 1986, **115**:47. doi:10.1111/j.1365-2133.1986.tb07700.x
6. Brown CA: **Incontinentia pigmenti: the development of pseudoglioma.** *Br J Ophthalmol* 1988, **72**:452–455.
7. Brunquell PJ: **Recurrent encephalomyelitis associated with incontinentia pigmenti.** *Pediatr Neurol* 1987, **3**:174–177.
8. Carney RG: **Incontinentia pigmenti. A world statistical analysis.** *Arch Dermatol* 1976, **112**:535–542.
9. Catalano RA, Lopatynsky M, Tasman WS: **Treatment of proliferative retinopathy associated with incontinentia pigmenti.** *Am J Ophthalmol* 1990, **110**:701–702.
10. Chabrolle JP, Le Luyer B: **Diagnostic différentiel d'une eruption cutanée d'étiologie non bactérienne chez le nouveauné: incontinentia pigmenti.** *Arch Fr Pediatr* 1986, **43**:219.
11. Cotterill JA: **Incontinentia pigmenti with associated nail changes.** *Br J Dermatol* 1987, **117**:49–50. doi:10.1111/j.1365-2133.1987.tb12045.x
12. Cramer W: **Incontinentia pigmenti. Bloch-Sulzberger syndrome.** *Tidsskr Nor Laegeforen* 1977, **97**:1678–1679.
13. Damstra RJ, Van Duren JA, Van Ginkel CWJ: **Incontinentia pigmenti (Bloch-Sulzberger).** *Br J Dermatol* 1991, **125**:280–281.
14. De Dulanto Campos C, Camacho-Martínez F: **Incontinentia pigmenti. Study of 7 cases, 2 in men.** *Ann Dermatol Venereol* 1979, **106**:337–343.
15. De Grouchy J, Turleau C, Doussau de Bazignan M, Maroteaux P, Thibaud D: **Incontinentia pigmenti (IP) and r(X). Tentative mapping of the IP locus to the X juxtacentromeric region.** *Ann Genet* 1985, **28**:86–89.
16. Diamantopoulos N, Bergman I, Kaplan S: **Actinomycosis meningitis in a girl with incontinentia pigmenti.** *Clinical Pediatrics* 1985, **24**:651–654.
17. Dolan OM, Bingham EA, Corbett JR: **Incontinentia pigmenti.** *Br J Dermatol* 1992, **127**:56–57. doi: 10.1111/j.1365-2133.1992.tb01241.x
18. Fellner MJ, Weinstein LH: **Incontinentia pigmenti in a boy.** *Int J Dermatol* 1978, **17**:67–68.
19. Fowell SM, Greenwald MJ, Prendiville JS, Jampol LM: **Ocular findings of incontinentia pigmenti in a male infant with Klinefelter syndrome.** *J Pediatr Ophthalmol Strabismus* 1992, **29**:180–184.
20. François J: **Incontinentia pigmenti (Bloch-Sulzberger syndrome) and retinal changes.** *Br J Ophthalmol* 1984, **68**:19–25.
21. Fried M, Meyer-Schwickerath GM: **Incontinentia pigmenti (Bloch-Sulzberger syndrome) associated with congenital retinal fold – a long-term 18-year observation.** *Klin Monatsbl Augenheilkd* 1980, **176**:44–49.
22. Fulton AB, Howard RO, Albert DM, Hsia YE, Packman S: **Ocular findings in triploidy.** *Am J Ophthalmol* 1977, **84**:859–867.
23. Garcia-Bravo B, Rodriguez-Pichardo A, Camacho-Martinez F: **Incontinentia pigmenti. Study of three families.** *Ann Dermatol Venereol* 1986, **113**:301–308.
24. García-Dorado J, de Unamuno P, Fernández-López E, Salazar Veloz J, Armijo M: **Incontinentia pigmenti: XXY male with a family history.** *Clin Genet* 1990, **38**:128–138.
25. Hashimoto K, Yoshikawa K: **A case of incontinentia pigmenti treated with short-term systemic corticosteroid.** *Skin* 1992, **34**:206–212.
26. Harman RR: **Incontinentia pigmenti.** *Br J Dermatol* 1977, **97**(Suppl 15):38–40.
27. Harris A, Collins J, Vetrie D, Cole C, Bobrow M: **X inactivation as a mechanism of selection against lethal alleles: further investigation of incontinentia pigmenti and X linked lymphoproliferative disease.** *J Med Genet* 1992, **29**:608–614.
28. Hauw JJ, Perié G, Bonnette J, Escourolle R: **Les 1ésions cérébrales de l'incontinentia pigmenti. A propos d'un cas anatomique.** *Acta Neuropathol* 1977, **38**:159–162. doi: 10.1007/BF00688564
29. Heathcote JG, Schoales BA, Willis NR: **Incontinentia pigmenti (Bloch-Sulzberger syndrome): a case report and review of the ocular pathological features.** *Can J Ophthalmol* 1991, **26**:229–237.

30. Hecht F, Hecht BK, Austin WJ: **Incontinentia pigmenti in Arizona Indians including transmission from mother to son inconsistent with the half chromatid mutation model.** *Clin Genet* 1982, **21**:293–296.
31. Highet AS: **Incontinentia pigmenti.** *Br J Dermatol* 1987, **117**:93–94. doi:10.1111/j.1365-2133.1987.tb12081.x
32. Himelhoch DA, Scott BJ, Olsen RA: **Dental defects in incontinentia pigmenti: case report.** Pediatr Dent 1987, **9**:236–239.
33. Hodgson SV, Neville B, Jones RW, Fear C, Bobrow M: **Two cases of X/autosome translocation in females with incontinentia pigmenti.** *Hum Genet* 1985, **71**:231–234.
34. Jain RB, Willetts GS: **Fundus changes in incontinentia pigmenti (Bloch-Sulzberger syndrome): a case report.** *Br J Ophthalmol* 1978, **62**:622–626.
35. Jain VK, Kalla G, Bumb RA: **Incontinentia pigmenti.** *Indian Dermatol Venerol Leprol* 1992, **58**:39–40.
36. Jessen RT, Van Epps DE, Goodwin JS, Bowerman J: **Incontinentia pigmenti. Evidence for both neutrophil and lymphocyte dysfunction.** *Arch Dermatol* 1978, **114**:1182–1186.
37. Kadotani T, Watanabe Y, Shimoda H: **A chromosome study of a patient with Wilms' tumor.** *Proc Jpn Acad Ser B* 1984, **60**:191–194.
38. Kajii T, Tsukahara M, Fukushima Y, Hata A, Matsuo K, Kuroki Y: **Translocation (X;13)(p11.21;q12.3) in a girl with incontinentia pigmenti and bilateral retinoblastoma.** *Ann Genet* 1985, **28**:219–223.
39. Kelly TE, Rary JM, Young L: **Incontinentia pigmenti: a chromosomal breakage syndrome.** *J Hered* 1976, **67**:171–172.
40. Korstanje MJ, Bessems PJ: **Incontinentia pigmenti with hyperkeratotic lesions in adulthood and possible squamous cell carcinoma.** *Dermatologica* 1991, **183**:234–236.
41. Kunze J, Frenzel UH, Hüttig E, Grosse F-R, Wiedemann H-R: **Klinefelter's syndrome and incontinentia pigmenti Bloch-Sulzberger.** *Hum Genet* 1977, **35**:237–240.
42. Kurczynski TW, Berns JS, Johnson WE: **Studies of a family with incontinentia pigmenti variably expressed in both sexes.** *J Med Genet* 1982, **19**:447–451.
43. Larsen R, Ashwal S, Peckham N: **Incontinentia pigmenti: association with anterior horn cell degeneration.** *Neurology* 1987, **37**:446–450.
44. Lenz W, Ullrich E, Witkowski R, Opitz C: **Unilateral Incontinentia pigmenti in a man.** *Padiatr Padol* 1982, **17**:187–199.
45. Manthey R, Apple DJ, Kivlin JD: **Iris hypoplasia in incontinentia pigmenti.** *J Pediatr Ophthalmol Strabismus* 1982, **19**:279–280.
46. Mascaro JM, Palou J, Vives P: **Painful subungual keratotic tumors in incontinentia pigmenti.** *J Am Acad Dermatol* 1985, **13**:913–918.
47. McKenna KE, Hughes AE, Allen G: **Incontinentia pigmenti.** *Br J Dermatol* 1992, **127**:55–56. doi:10.1111/j.1365-2133.1992.tb01240.x
48. Menni S, Piccinno R, Biolchini A, Plebani A: **Immunologic investigations in eight patients with incontinentia pigmenti.** *Pediatr Dermatol* 1990, **7**:275–277.
49. Migeon BR, Axelman J, Jan de Beur S, Valle D, Mitchell GA, Rosenbaum KN: **Selection against lethal alleles in females heterozygous for incontinentia pigmenti.** *Am J Hum Genet* 1989, **44**:100–106.
50. Moss C, Ince P: **Anhidrotic and achromians lesions in incontinentia pigmenti.** *Br J Dermatol* 1987, **116**:839–849.
51. Narbona E, Bayes R, Maldonado J, Naranjo R: **Incontinentia pigmenti. Study of a male in the initial stage.** *An Esp Pediatr* 1982, **17**:482–484.
52. Nazzaro V, Brusasco A, Gelmetti C, Ermacora E, Caputo R: **Hypochromic reticulated streaks in incontinentia pigmenti: an immunohistochemical and ultrastructural study.** *Pediatr Dermatol* 1990, **7**:174–178.
53. Nix RR, Apple DJ: **Proliferative retinopathy associated with incontinentia pigmenti.** *Retina* 1981, **1**:156–161.
54. O'Brien JE, Feingold M: **Incontinentia pigmenti. A longitudinal study.** *Am J Dis Child* 1985, **139**:711–712.
55. Olumide Y, Danesi M, McMoli T: **Incontinentia pigmenti in Nigerians.** *Int J Dermatol* 1983, **22**:419–421.
56. Ormerod AD, White MI, McKay E, Johnston AW: **Incontinentia pigmenti in a boy with Klinefelter's syndrome.** *J Med Genet* 1987, **24**:439–441.
57. Pallotta R, Dalprá L: **Chromosomal instability in incontinentia pigmenti: study of four families.** *Ann Genet* 1988, **31**:27–31.
58. Parcheta B, Skawinski W, Wisniewski L, Piontek E, Ryzko J: **Clinical features and computer-aided analysis of chromosome aberration in a case with incontinentia pigmenti.** *Klin Padiatr* 1987, **199**:32–36.
59. Peltonen L: **Incontinentia pigmenti in four generations.** *Dermatologica* 1986, **172**:201–204.

60. Petri H, Pierchalla P, Tronnier H: **Bloch-Sulzberger incontinentia pigmenti with associated neurologic and ophthalmologic complications.** *Z Hautkr* 1988, **63**:568–572.
61. Pongprasit P, Chittinand S, Lerchawanakul A, Chermsiriwat S: **Incontinentia pigmenti, analysis of 15 cases.** *J Med Assoc Thai* 1985, **68**:630–637.
62. Rahi J, Hungerford J: **Early diagnosis of the retinopathy of incontinentia pigmenti: successful treatment by cryotherapy.** *Br J Ophthalmol* 1990, **74**:377–379.
63. Roberts WM, Jenkins JJ, Moorhead EL 2nd, Douglass EC: **Incontinentia pigmenti, a chromosomal instability syndrome, is associated with childhood malignancy.** *Cancer* 1988, **62**:2370–2372.
64. Rosenfeld SI, Smith ME: **Ocular findings in incontinentia pigmenti.** *Ophthalmology* 1985, **92**:543–546.
65. Sasaki M, Hanaoka S, Suzuki H, Takashima S, Arima M: **Cerebral white matter lesions in a case of incontinentia pigmenti with infantile spasms, mental retardation and left hemiparesis.** *No To Hattatsu* 1991, **23**:278–283.
66. Schaller J, Kock M, Goos M: **Onset of disseminated Bloch-Sulzberger incontinentia pigmenti**. *Hautarzt* 1992, **43**:383–385.
67. Shuper A, Bryan RN, Singer HS: **Destructive encephalopathy in incontinentia pigmenti: A primary disorder?** *Pediatr Neurol* 1990, **6**:137–140.
68. Siemes H, Schneider H, Dening D, Hanefeld F: **Encephalitis in two members of a family with incontinentia pigmenti (Bloch-Sulzberger syndrome). The possible role of inflammation in the pathogenesis of CNS involvement.** *Eur J Pediatr* 1978, **129**:103–115.
69. Simmons DA, Kegel MF, Scher RK, Hines YC: **Subungual tumors in incontinentia pigmenti.** *Arch Dermatol* 1986, **122**:1431–1434.
70. Smith B, Bedrossian EH Jr: **Incontinentia pigmenti associated with nasolacrimal duct obstruction.** *Ophthalmic Surg* 1984, **15**:980–982.
71. Sommer A, Liu PH: **Incontinentia pigmenti in a father and his daughter.** *Am J Med Genet* 1984, **17**:655–659.
72. Spallone A: **Incontinentia pigmenti (Bloch-Sulzberger syndrome): seven case reports from one family.** *Br J Ophthalmol* 1987, **71**:629–634.
73. Takematsu H, Terui T, Torinuki W, Tagami H: **Incontinentia pigmenti: eosinophil chemotactic activity of the crusted scales in the vesiculobullous stage.** *Br J Dermatol* 1986, **115**:61–66.
74. Tanaka K, Kambe N, Fujita M, Ando Y, Takashima S, Yuasa I: **Incontinentia pigmenti in identical twins with separate skin and neurological disorders.** *Acta Derm Venerol* 1990, **70**:267–268.
75. Thyresson NH, Goldberg NC, Tye MJ, Leiferman KM: **Localization of eosinophil granule major basic protein in incontinentia pigmenti.** *Pediatr Dermatol* 1991, **8**:102–106.
76. Tidman MJ, Neville BGR, Wells RS: **Coexistent incontinentia pigmenti and tuberous sclerosis.** *Br J Dermatol* 1985, **113**:52. doi:10.1111/j.1365-2133.1985.tb13022.x
77. Triki C, Devictor D, Kah S, Roge-Wolter M, Lacroix C, Venencie Py, Landrieu P: **Cerebral complications of incontinentia pigmenti. A clinicopathological study of a case.** *Rev Neurol (Paris) 1992*, **148**:773–776.
78. Wiklund DA, Weston WL: **Incontinentia pigmenti. A four-generation study.** *Arch Dermatol* 1980, **116**:701–703.
79. Worret WI, Nordquist RE, Burgdorf WH: **Abnormal cutaneous nerves in incontinentia pigmenti.** *Ultrastruct Pathol* 1988, **12**:449–454. doi:10.3109/01913128809064214
80. Yell JA, Walshe M, Desai SN: **Incontinentia pigmenti associated with bilateral cleft lip and palate.** *Clin Exp Dermatol* 1991, **16**:49–50.
81. Yu YS, Cho BJ: **Cryotherapy for retinopathy of incontinentia pigmenti.** *Korean J Ophthalmol* 1991, **5**:47–50
82. Zillikens D, Mehringer A, Lechner W, Burg G: **Hypo- and hyperpigmented areas in incontinentia pigmenti. Light and electron microscopic studies.** *Am J Dermatopathol* 1991, **13**:57–62.
