# Supplementary material for: Systematic review of central nervous system anomalies in incontinentia pigmenti
Source: Orphanet J Rare Dis. 2013 Feb 13;8:25. doi: 10.1186/1750-1172-8-25 (PMC3576363; doi:10.1186/1750-1172-8-25)
Supplement: Additional file 2 — Additional references published in the period of 1993-2012. [file 1750-1172-8-25-S2.pdf]

Systematic review of central nervous system anomalies in incontinentia pigmenti

Snežana Minić, Dušan Trpinac, Miljana Obradović

S. Minić
School of Medicine, University of Belgrade, and
Dermatovenerology Clinic, Clinical Center of Serbia,
Deligradska 34, 11000 Belgrade, Serbia
e-mail: dtrpinac@eunet.rs
Tel.: +381-64-199-88-67
Fax: +381-11-361-25-67

Additional file 2

Additional references published in the period of 1993-2012

1. Abadjieva T: **Incontinentia pigmenti.** *J Eur Acad Dermatol Venereol* 2004, **18**:325.
2. Abe S, Okumura A, Hamano SI, Tanaka M, Shiihara T, Aizaki K, Tsuru T, Toribe Y, Arai H, Shimizu T: **Early infantile manifestations of incontinentia pigmenti mimicking acute encephalopathy.** *Brain Dev* 2011, **33**:28–34. doi:10.1016/j.braindev.2010.04.002
3. Abimelec P, Rybojad M, Cambiaghi S, Moraillon I, Cavelier-Balloy B, Marx C, Morel P: **Late, painful, subungual hyperkeratosis in incontinentia pigmenti.** *Pediatr Dermatol* 1995, **12**:340–342.
4. Adeniran A, Townsend PL, Peachey RD: **Incontinentia pigmenti (Bloch-Sulzberger syndrome) manifesting as painful periungual and subungual tumours.** *J Hand Surg (Br)* 1993, **18**:667–669.
5. Agarwal P: **Incontinentia pigmenti.** *Indian J Dermatol Venereol Leprol* 1997, **63**:368–369.
6. Aggarwal K, Jain VK, Dayal S: **Incontinentia pigmenti with nail dystrophy.** *Indian J Dermatol Venereol Leprol* 2003, **69**:3–4.
7. Albernaz VS, Castillo M, Hudgins PA, Mukherji SK: **Imaging findings in patients with clinical anophthalmos.** *AJNR Am J Neuroradiol* 1997, **18**:555–561.
8. Alle D, Venglarcik J, Williams D, Casanova J, Hostoffer Jr. R: **Mutation of *NEMO* associated with neutrophil chemotaxis abnormality.** *J Allergy Clin Immunol* 2006, **117**:S291.
9. Allison CL, Sigler MK: **Ocular findings in incontinentia pigmenti.** *Optometry* 2008, **79**:320.
10. Alikhan A, Lee AD, Swing D, Carroll C, Yosipovitch G: **Vaccination as a probable cause of incontinentia pigmenti reactivation.** *Pediatr Dermatol* 2010, **27**:62–64. doi:10.1111/j.1525-1470.2009.01047.x
11. Al-Khenaizan S: **Incontinentia pigmenti.** *Ann Saudi Med* 2000, **20**:424–426.
12. Al-Zuhaibi S, Ganesh A, Al-Waili A, Al-Azri F, Javad H, Al-Futaisi A: **A female child with skin lesions and seizures.** *SQU Med J* 2009, **9**:157–161.
13. Amaya M, Honda Y, Yamashiro M: **A case of mild type of incontinentia pigmenti.** *Nishinihon J Dermatol* 2002, **64**:570–573.
14. Ardelean D, Pope E: **Incontinentia pigmenti in boys: a series and review of the literature.** *Pediatr Dermatol* 2006, **23**:523–527.
15. Arenas-Sordo Mde L, Vallejo-Vega B, Hernández-Zamora E, Gálvez-Rosas A, Montoya-Pérez LA: **Incontinentia pigmenti (IP2) familiar case report with affected men. Literature review.** *Med Oral Patol Oral Cir Bucal* 2005, **10**:E122–129.
16. Astle WF, Papp A, Huang PT, Ingram A: **Refractive laser surgery in children with coexisting medical and ocular pathology.** *J Cataract Refract Surg* 2006, **32**:103–108. doi:10.1016/j.jcrs.2005.11.028
17. Awadh M, O'Regan ME: **Incontinentia pigmenti: rare differential diagnosis in neonatal seizures with acute skin eruptions.** *Eur J Pediatr Neurol* 2009, **13**(Suppl 1):S81.
18. Aydingöz U, Midia M: **Central nervous system involvement in incontinentia pigmenti: cranial MRI of two siblings.** *Neuroradiology* 1998, **40**:364–366.
19. Bachevalier F, Marchal C, Di Cesare MP, Antunes A, Truchetet F: **Atteinte neurologique létale au cours d'une incontinentia pigmenti.** *Ann Dermatol Venereol* 2003, **130**:1139–1142. doi:AD-12-2003-130-12-0151-9638-101019-ART8
20. Balaratnasingam C, Lam GC: **Retinal sequelae of incontinentia pigmenti.** *Pediatr Int* 2009, **51**:141–143. doi:10.1111/j.1442-200X.2008.02780.x
21. Bandello F, Incorvaia C, Parmeggiani F, D'Angelo S, Costagliola C, Sebastiani A: **Management of incontinentia pigmenti: a case of monolateral preretinal fibrovascular proliferations adjacent to snail-track degeneration areas.** *Eur J Ophthalmol* 2002, **12**:339–342.

22. Bardaro T, Falco G, Sparago A, Mercadante V, Gean Molins E, Tarantino E, Ursini MV, D'Urso M: **Two cases of misinterpretation of molecular results in incontinentia pigmenti, and a PCR-based method to discriminate *NEMO/IKKγ* gene deletion.** *Hum Mutat* 2003, **21**:8–11. doi:10.1002/humu.10150
23. Barkham M, Ogboli M, Moss C: **Late recurrent inflammation of incontinentia pigmenti lesions coinciding with acute infection.** *J Am Acad Dermatol* 2007, **56**:AB158.
24. Basarab T, Dunnill MG, Munn SE, Russell-Jones R: **Incontinentia pigmenti: variable disease expression within an affected family.** *J Eur Acad Dermatol Venereol* 1998, **11**:173–176.
25. Batioglu F, Özmert E: **Early indirect laser photocoagulation to induce regression of retinal vascular abnormalities in incontinentia pigmenti.** *Acta Ophthalmol* 2010, **88**:267–268. doi:10.1111/j.1755-3768.2008.01394.x
26. Bell WR, Green WR, Goldberg MF: **Histopathologic and trypsin digestion studies of the retina in incontinentia pigmenti.** *Ophthalmology* 2008, **115**:893–897. doi:10.1016/j.ophtha.2007.08.027
27. Beni SM, Hoffmann C, Goshen E, Friedman M, Ben-Zeev B: **Stroke-like episodes in rare neurocutaneous syndromes.** *Neuropediatrics* 2006. doi:10.1055/s-2006-943681
28. Bentolila R, Rivera H, Sanchez-Quevedo MC: **Incontinentia pigmenti: a case report.** *Pediatr Dent* 2006, **28**:54–57.
29. Benyelles N, Uteza Y, Bodemer C, Roche O, Dureau P, Dufier JL: **Ocular findings of Incontinentia pigmenti.** *J Fr Ophtalmol* 2002, **25**:165.
30. Berinstein DM, Trese MT: **Anomalous foveal vascular pattern in a case of incontinentia pigmenti.** *Retina* 1999, **19**:564–566.
31. Bharti R, Bal SM: **Incontinentia pigmenti.** *Indian Dermatol Venerol Leprol* 1995, **58**:39–40.
32. Birusingh RJ, Siddiqui R, Kilchevsky ES: **Surviving male newborn with incontinentia pigmenti.** *Arch Pathol Lab Med* 2006, **130**:1372.
33. Bittar M, Danarti R, Konig A, Gal A, Happle R: **Late-onset familial onychodystrophy heralding incontinentia pigmenti.** *Acta Derm Venereol* 2005, **85**:274–275. doi:10.l080/000l5550410026l46
34. Bodak N, Hadj-Rabia S, Hamel-Teillac D, de Prost Y, Bodemer C: **Late recurrence of inflammatory first-stage lesions in incontinentia pigmenti: an unusual phenomenon and a fascinating pathologic mechanism.** *Arch Dermatol* 2003, **139**:201–204.
35. Bonnekoh B, Dippel E, Franke I, Kolesnik M, Jakubiczka S, Orth U, Muschke P, Gollnick H, Gal A**: Incontinentia pigmenti (Bloch-Sulzberger) in inflammatory bullous stage without associated malformations and with evidence of c.723C>G mutation in the *NEMO* gene.** *J Dtsch Dermatol Ges* 2009, **7**:244.
36. Boor SB, Vucurevic G, Boor R, Kutschke G, Stoeter P: **Bildgebung, DTI und Spektroskopie bei Incontinentia pigmenti (Bloch-Sulzberger-Syndrom).** *Fortschr Röntgenstr* 2004. doi:10.1055/s-2004-828208
37. Boutli F, Tsakiri A, Kogia M, Karakatsanis G, Mourellou O: **Incontinentia pigmenti.** *J Eur Acad Dermatol Venereol* 2003, **17**:277.
38. Bryant SA, Rutledge SL: **Abnormal white matter in a neurologically intact child with incontinentia pigmenti.** *Pediatr Neurol* 2007, **36**:199–201. doi:10.1016/j.pediatrneurol.2006.11.009
39. Buchanan JAG, Atkin PA, Davison S, Leonard JN, Gregor ZJ, Webster A, Thornhill MH: **Delayed presentation of incontinentia pigmenti.** *J Oral Pathol Med* 2000, **29**:464.
40. Buinauskaite E, Buinauskiene J, Kucinskiene V, Strazdiene D, Valiukeviciene S: **Incontinentia pigmenti in a male infant with Klinefelter syndrome: a case report and review of the literature.** *Pediatr Dermatol* 2010, **27**:492–495. doi:10.1111/j.1525-1470.2010.01261.x
41. Buinauskiene J, Buinauskaite E, Valiukeviciene S: **Incontinentia pigmenti (Bloch-Sulzberger syndrome) in neonates.** *Medicina (Kaunas)* 2005, **41**:496–499.
42. Burkhardt D, Schuffenhauer S, Peter RU, Walther JU, Ruzicka T: **Incontinentia pigmenti in a male patient.** *Hautarzt* 1993, **44**:153–156.
43. Bussade M, Esposti A, Roquini J, Rutowitsch M: **Incontinentia pigmenti (Bloch-Sulzberger syndrome).** *J Am Acad Dermatol* 2005, **52**:P110.
44. Carrascosa Romero MC, Ruiz Cano R, Medina Monzon C, Perez Garcia L, Martinez Gutierrez A, Tebar Gil R: **Neonatal convulsions caused by incontinentia pigmenti with left opercular dysgenesia.** *Rev Neurol* 2003, **36**:36–39.
45. Cartwright MS, White DL, Miller III LM, Roach ES. **Recurrent stroke in a child with incontinentia pigmenti.** *J Child Neurol* 2009, **24**:603–605. doi:10.1177/0883073808327835
46. Cates CA, Dandekar SS, Flanagan DW, Moore AT: **Retinopathy of incontinentia pigmenti: a case report with thirteen years follow-up.** *Ophthalmic Genet* 2003, **24**:247–252.
47. Cerruto CA, Pichardo RO, Sangueza OP: **Incontinentia pigmenti: a presentation of four cases and literature review.** *Am J Dermatopathol* 2005, **27**:533. doi:10.1097/01.dad.0000197731.79791.75
48. Chan YC, Giam YC: **A retrospective study of incontinentia pigmenti seen at the National Skin Centre, Singapore over a 10-year period.** *Ann Acad Med Singapore* 2001, **30**:409–413.
49. Chan YC, Happle R, Giam YC: **Whorled scarring alopecia: a rare phenomenon in incontinentia pigmenti?** *J Am Acad Dermatol* 2003, **49**:929–931. doi:10.1067/S0190-9622(03)00474-2
50. Chang JT, Chiu PC, Chen YY, Wang HP, Hsieh KS: **Multiple clinical manifestations and diagnostic challenges of incontinentia pigmenti – 12 years' experience in 1 medical center.** *J Chin Med Assoc* 2008, **71**:455–460.

51. Chang T, Behshad R, Brodell B, Gilliam A: **A male infant with anhidrotic ectodermal dysplasia/immunodeficiency accompanied by incontinentia pigmenti and a mutation in the *NEMO* pathway.** *J Am Acad Dermatol* 2008, **58**:316–320. doi:10.1016/j.jaad.2007.02.024
52. Chatkupt S, Gozo AO, Wolansky LJ, Sun S: **Characteristic MR findings in a neonate with incontinentia pigmenti.** *AJR Am J Roentgenol* 1993, **160**:372–374.
53. Cho MJ, Shin SM, Moon HK: **A case of incontinentia pigmenti developed in a male newborn infant.** *Yeungnam Univ J Med* 1998, **15**:381–390.
54. Cho SY, Lee CK, Drummond BK: **Surviving male with incontinentia pigmenti: a case report.** *Int J Paediatr Dent* 2004, **14**:69–72.
55. Chujo S, Hayakawa I, Hamaguchi Y, Shinozaki M, Kawara S, Okuda N, Inaoki M: **A case of incontinentia pigmenti with retinal vascular abnormality and left cerebral atrophy.** *Jpn J Clin Dermatol* 2005, **59**:54–56.
56. Chung WK, Lee DW, Chang SE, Lee MW, Choi JH, Moon KC: **A case of incontinentia pigmenti associated with multiorgan abnormalities.** *Ann Dermatol (Seoul)* 2009, **21**:56–59.
57. Ciarallo L, Paller AS: **Two cases of incontinentia pigmenti simulating child abuse.** *Pediatrics* 1997, **100**:E6.
58. Clemons E, Clemons D, Lee JA, Berney S: **Incontinentia pigmenti in three generations: a case report.** *J Am Acad Dermatol* 2008, **58**(Suppl 2):AB80.
59. Cohen PR: **Incontinentia pigmenti: clinicopathologic characteristics and differential diagnosis.** *Cutis* 1994, **54**:161–166.
60. Coleman R, Genet SA, Harper JI, Wilkie AO: **Interaction of incontinentia pigmenti and factor VIII mutations in a female with biased X inactivation, resulting in haemophilia.** *J Med Genet* 1993, **30**:497–500.
61. Čulić V, Gabrić D, Puizina-Ivić N, Rozman K, Peterlin B, Pavelić J: **De novo *NEMO* gene deletion (delta4-10) – a cause of incontinentia pigmenti in a female infant: a case report.** *Coll Antropol* 2008, **32**:1259–1262.
62. Daikoku N, Higuchi M, Miyagawa S: **A case of incontinentia pigmenti observed from the early inflammatory stage at birth.** *Skin Research* 2002, **1**:190–193.
63. Darné S, Carmichael AJ: **Isolated recurrence of vesicobullous incontinentia pigmenti in a schoolgirl.** *Br J Dermatol* 2007, **156**:600–602. doi:10.1111/j.1365-2133.2006.07700.x
64. De Argila D, Rivera R, Guerra A, Iglesias L: **Incontinentia pigmenti: a case with an unusual course.** *Pediatr Dermatol* 1996, **13**:434–435.
65. Defazio J, Powers R: **Incontinentia pigmenti (IP) with congenital nasolacrimal duct obstruction: a case report and review.** *J Am Acad Dermatol* 2007, **56**:AB111.
66. Demirel N, Aydin M, Zenciroglu A, Okumus N, Tekgunduz KS, Ipek MS, Boduroglu E: **Incontinentia pigmenti with encephalocele in a neonate: a rare association.** *J Child Neurol* 2009, **24**:495–499. doi:10.1177/0883073808324774
67. DeVetten G, Ells A: **Fluorescein angiographic findings in a male infant with incontinentia pigmenti.** *J AAPOS* 2007, **11**:511–512. doi:10.1016/j.jaapos.2007.03.006
68. Devriendt K, Matthijs G, Fryns JP, Ballegeer V: **Second trimester miscarriage of a male fetus with incontinentia pigmenti.** *Am J Med Genet* 1998, **80**:298–299.
69. Di Landro A, Marchesi L, Reseghetti A, Cainelli T: **Warty linear streaks of the palm and sole: possible late manifestations of incontinentia pigmenti.** *Br J Dermatol* 2000, **143**:1102–1103.
70. Doblhofer M, Willerding G, Ottenberg D, Bechrakis NE, Jandeck C, MH Foerster MH**: 11 patients with ocular involvement in incontinentia pigmenti.** *Klin Monatsbl Augenheilkd* 2009. doi:10.1055/s-0029-1243718
71. Domagała A, Wojtowicz-Prus E, Woźniak Z, Węgłowska J: **Incontinentia pigmenti in a neonate – a case report.** *Derm Klin* 2008, **10**:155–158.
72. Domínguez-Reyes A, Aznar-Martin T, Cabrera-Suarea E: **General and dental characteristics of Bloch-Sulzberger syndrome.** Review of literature and presentation of a case report. *Med Oral* 2002, **7**:293–297.
73. Donati P, Muscardin L, Amantea A, Paolini F, Venuti A: **Detection of HPV-15 in painful subungual tumors of incontinentia pigmenti: successful topical therapy with retinoic acid.** *Eur J Dermatol* 2009, **19**:243–247.
74. Doruk C, Bicakci AA, Babacan H: **Orthodontic and orthopedic treatment of a patient with incontinentia pigmenti.** *Angle Orthod* 2003, **73**:763–768.
75. Dufke A, Vollmer B, Kendziorra H, Mackensen-Haen S, Orth U, Orlikowsky T, Gal A: **Hydrops fetalis in three male fetuses of a female with incontinentia pigmenti.** *Prenat Diagn* 2001, **21**:1019–1021. doi:10.1002/pd.165
76. Dupuis-Girod S, Corradini N, Hadj-Rabia S, Fournet JC, Faivre L, Le Deist F, Durand P, Döffinger R, Smahi A, Israel A, Courtois G, Brousse N, Blanche S, Munnich A, Fischer A, Casanova JL, Bodemer C: **Osteopetrosis, lymphedema, anhidrotic ectodermal dysplasia, and immunodeficiency in a boy and incontinentia pigmenti in his mother.** *Pediatrics* 2002, **109**:e97.
77. Dutheil P, Vabres P, Cayla MC, Enjolras O: **Incontinentia pigmenti: late sequelae and genotypic diagnosis: a three-generation study of four patients.** *Pediatr Dermatol* 1995, **12**:107–111.
78. El Fekih L, Hmaied W, Souissi K, Nasri H, Derbel F, Hamdi A: **Incontinentia pigmenti: a rare cause of retinal vasculitis in children.** *Tunis Med* 2008, **86**:1079–1108.
79. Emery MM, Siegfried EC, Stone MS, Stone EM, Patil SR: **Incontinentia pigmenti: transmission from father to daughter.** *J Am Acad Dermatol* 1993, **29**:368–372.
80. Emre S, Fırat Y, Güngör S, Fırat AK, Yelda Karıncaoğlu Y: **Incontinentia pigmenti: a case report and literature review.** *Turk J Pediatr* 2009, **51**:190–194.

81. Enei LG, Orllana IA, Vargas XR, Salazar RC, Paschoal F: **Mother and daughter incontinentia pigmenti. Case report.** *Rev Chil Pediatr* 2011, **82**:225–230. doi:10.4067/S0370-41062011000300008
82. Equi R, Bains HS, Jampol LM, Goldberg MF: **Retinal tears occurring at the border of vascular and avascular retina in adult patients with incontinentia pigmenti.** *Retina* 2003, **23**:574–576.
83. Escobedo J: **Incontinentia pigmenti without systemic malformations: a case report and description for primary care clinicians.** *Proceedings of UCLA Healthcare* 2000, **4**:10–22.
84. Escudero J, Borras F, Fernández MA, Domínguez C: **Fluorescein angiography with Retcam in incontinentia pigmenti: a case report.** *Arch Soc Esp Oftalmol* 2009, **84**:529–532.
85. Esholdt IH: **Incontinentia pigmenti.** *Nord Med* 1994, **109**:58–60.
86. Fairhurst D, Sommer S, Clark SM: **A subungual tumour in a 37-year-old woman: an unusual presentation of incontinentia pigmenti.** *Br J Dermatol* 2008, **159**(Suppl. 1):20.
87. Faloyin M, Levitt J, Bercowitz E, Carrasco D, Tan J: **All that is vesicular is not herpes: incontinentia pigmenti masquerading as herpes simplex virus in a newborn.** *Pediatrics* 2004, **114**:e270–272. doi:10.1542/peds.114.2.e270
88. Fard AK, Goldberg MF: **Persistence of fetal vasculature in the eyes of patients with incontinentia pigmenti.** *Arch Ophthalmol* 1998, **116**:682–684.
89. Feito-Rodríguez M, García-Macarrón J, Bravo-Burguillos ER, Vera-Casaño A, de Lucas-Laguna R: **Incontinentia pigmenti: three new cases that demonstrate it is not only a matter of women.** *Actas Dermosifiliogr* 2007, **98**:112–115.
90. Fekrat S, Humayun MS, Goldberg MF: **Spontaneous retinal reattachment in incontinentia pigmenti.** *Retina* 1998, **18**:75–77.
91. Fernández MF, Samela PC, Buján MM, Merediz J, Pierini AM: **Neonate with linear rows of vesiculobullous lesions.** *Arch Argent Pediatr* 2010, **108:**e5–8.
92. Ferreira RC, Ferreira LC, Forstot L, King R: **Corneal abnormalities associated with incontinentia pigmenti.** *Am J Ophthalmol* 1997, **123**:549–551.
93. Ferreira RC, Shea C, Johnson DW, Bateman JB: **Electroretinography in incontinentia pigmenti.** *J AAPOS* 1997, **1**:172–174.
94. Fiorillo L, Sinclair DB, O'Byrne ML, Krol AL: **Bilateral cerebrovascular accidents in incontinentia pigmenti.** *Pediatr Neurol* 2003, **29**:66–68. doi:10.1016/S0887-8994(03)00144-9
95. Franco LM, Goldstein J, Prose NS, Selim MA, Tirado CA, Coale MM, McDonald MT: **Incontinentia pigmenti in a boy with XXY mosaicism detected by fluorescence in situ hybridization.** *J Am Acad Dermatol* 2006, **55**:136–138. doi:10.1016/j.jaad.2005.11.1068
96. Fromer ES, Lynch PJ: **Neonatal herpes simplex and incontinentia pigmenti.** *Pediatr Dermatol* 2001, **18**:86–87.
97. Fryssira H, Kakourou T, Valari M, Stefanaki K, Amenta S, Kanavakis E: **Incontinentia pigmenti revisited. A novel nonsense mutation of the *IKBKG* gene.** *Acta Paediatr* 2011, **100**:128–133. doi:10.1111/j.1651-2227.2010.01921.x
98. Fu LW, Soong WJ, Tsai SC, Hwang B: **Retinopathy in incontinentia pigmenti: a neonatal case report.** *Zhonghua Min Guo Xiao Er Ke Yi Xue Hui Za Zhi* 1995, **36**:210–213.
99. Fujii K, So H, Ogino H, Fujiwara T, Tanabe T, Okamoto S, Yuge K, Imazumi M: **Incontinentia pigmenti in four generations.** *Skin Research* 2004, **3**:378–383.
100. Fusco F, Bardaro T, Fimiani G, Mercadante V, Miano MG, Falco G, Israël A, Courtois G, D'Urso M, Ursini MV: **Molecular analysis of the genetic defect in a large cohort of IP patients and identification of novel *NEMO* mutations interfering with NF-κB activation.** *Hum Mol Genet* 2004, **13**:1763–1773. doi:10.1093/hmg/ddh192
101. Fusco F, Fimiani G, Tadini G, Michele D, Ursini MV: **Clinical diagnosis of incontinentia pigmenti in a cohort of male patients.** *J Am Acad Dermatol* 2007, **56**:264–267. doi:10.1016/j.jaad.2006.09.019
102. Gajić-Veljić M, Nikolić MM, Mijušković M: **Incontinentia pigmenti – three cases without serous systemic involvement.** *J Eur Acad Dermatol Venereol* 2004, **18**:321.
103. Garzón AE, Garzón VH, Gallegos X: **Male child patient with incontinentia pigmenti.** *Pediat Dermatol Suppl* 2001, **18**:96.
104. Ghaffar SA, Stephen J, Brotherton H, Benton C, Schofield O: **Varied presentations of incontinentia pigmenti.** Arch Dis Child 2010, **95**:A15. doi:10.1136/adc.2010.186338.34
105. Godambe S, McNamara P, Rajguru M, Hellmann J: **Unusual neonatal presentation of incontinentia pigmenti with persistent pulmonary hypertension of the newborn: a case report.** *J Perinatol* 2005, **25**:289–292. doi:10.1038/sj.jp.7211250
106. Goldberg MF: **The blinding mechanisms of incontinentia pigmenti.** *Trans Am Ophthalmol Soc* 1994, **92**:167–176.
107. Goldberg MF: **Macular vasculopathy and its evolution in incontinentia pigmenti.** *Ophthalmic Genet* 1998, **19**:141–148.
108. Goldberg MF, Custis PH: **Retinal and other manifestations of incontinentia pigmenti (Bloch-Sulzberger syndrome).** *Ophthalmology* 1993, **100**:1645–1654.
109. González Burgos L, Di Martino Ortiz B, Rodríguez Masi M, Knopfelmacher O, Bolla de Lezcano L**: Bloch-Sulzberger's syndrome (Incontinentia pigmenti). Contribution with a case report.** *Arch Argent Pediatr* 2011, **109**:e62–65.

110. Griesinger G, Büdgen N, Salmen D, Schwinger E, Gillessen-Kaesbach, Diedrich K: **Polar body biopsy in the diagnosis of monogenic diseases: the birth of three healthy children.** *Dtsch Arztebl Int* 2009, **106**:533–538. doi:10.3238/arztebl.2009.0533
111. Hadj-Rabia S, Froidevaux D, Bodak N Hamel-Teillac D, Smahi A, Touil Y, Fraitag S, de Prost Y, Bodemer C: **Clinical study of 40 cases of incontinentia pigmenti.** *Arch Dermatol* 2003, **139**:1163–1170. doi:10.1001/archderm.139.9.1163
112. Haga T: **Incontinentia pigmenti with deformity of ocular lesion.** *Rinsho Derma (Tokyo)* 2006, **48**:749–751.
113. Haller-Kikkatalo K, Peters M, Kisand K, Soritsa A, Reimand T, Salumets A: **Incontinentia pigmenti in a female conceived by in vitro fertilization.** *Am J Med Genet Part A* 2008, **146A**:3092–3094. doi:10.1002/ajmg.a.32565
114. Han JK, Choi JC, Park MK,Park KW, Eun BL, Chung JT, Lee DH: **A case of incontinentia pigmenti with destructive encephalopathy.** *J Kor Neurol Ass* 1998, **16**:739–742.
115. Hart A, Edwards C, Mahajan J, Wood ML, Griffiths P: **Destructive encephalopathy in incontinentia pigmenti**. *Dev Med Child Neurol* 2009, **51**:162–163. doi:10.1111/j.1469-8749.2008.03121.x
116. Has C, Danescu S, Volz A, Nöh F, Technau K, Bruckner-Tuderman L: **Incontinentia pigmenti in a newborn with a novel nonsense mutation in the *NEMO* gene.** *Br J Dermatol* 2007, **156**:392–393. doi:10.1111/j.1365-2133.2006.07649.x
117. Hayes IM, Varigos G, Upjohn EJ, Orchard DC, Penny DJ, Savarirayan R: **Unilateral acheiria and fatal primary pulmonary hypertension in a girl with incontinentia pigmenti.** *Am J Med Genet A* 2005, **135A**:302–303. doi:10.1002/ajmg.a.30698
118. Hegde SK, Bhat SS, Soumya S, Pai D: **Incontinentia pigmenti.** *J Indian Soc Pedod Prev Dent* 2006, **24**:S24–26.
119. Hennel SJ, Ekert PG, Volpe JJ, Inder TE**: Insights into the pathogenesis of cerebral lesions in incontinentia pigmenti.** *Pediatr Neurol* 2003, **29**:148–150. doi:10.1016/S0887-8994(03)00150-4
120. Hershberger DH, Minnal V, Reynolds JD, Reidy JJ: **Isolated corneal findings in incontinentia pigmenti.** *J Pediatr Ophthalmol Strabismus* 2009, **46**:381. doi:10.3928/0191393-20091104-13
121. Hicks MI, Pride H, Ferringer T: **Keratoacanthoma developing within a hyperpigmented patch in a patient with incontinentia pigmenti.** *J Am Acad Dermatol* 2010, **62**(Suppl 1):AB112.
122. Hirano T, Torii Y, Seno A, Ueno Y, Tanahashi T: **Incontinentia pigmenti with ophthalmopathy.** *Rinsho Derma (Tokyo)* 2005, **47**:1111–1113.
123. Holmström G, Bergendal B, Hallberg G, Marcus S, Hallén A, Dahl N: **Incontinentia pigmenti** – **a rare disease with many symptoms.** *Läkartidningen* 2002, **99**:1345–1350.
124. Hsiao PF, Lin SP, Chiang SS, Wu YH, Chen HC, Lin YC: ***NEMO* gene mutations in Chinese patients with incontinentia pigmenti.** *J Formos Med Assoc* 2010, **109**:192–200. doi:10.1016/S0929-6646(10)60042-3
125. Huang J, Kondo H, Uchio E: A case of incontinentia pigmenti in Japan and its genetic examination. *Jpn J Ophthalmol* 2007, **51**:142–145. doi:10.1007/s10384-006-0412-3
126. Hubert JN, Callen JP: **Incontinentia pigmenti presenting as seizures.** Pediatr Dermatol 2002, **19**:550–552.
127. Hung PC, Wang HS: **Leukoencephalopathy: unusual sonographic finding in a neonate with incontinentia pigmenti.** *J Ultrasound Med* 2010, **29**:851–854.
128. Huttner HB, Richter G, Jünemann A, Kress W, Weis J, Schröder JM, Gal A, Doerfler A, Udd B, Schröder R: **Incontinetia pigmenti-related myopathy or unsolved ''double trouble''?** Neuromuscul Disord 2010, **20**:139–141. doi:10.1016/j.nmd.2009.12.006
129. Hydén-Granskog C, Salonen R, von Koskull H: **Three Finnish incontinentia pigmenti (IP) families with recombinations with the IP loci at Xq28 and Xp11.** *Hum Genet* 1993, **91**:185–189.
130. Inostroza MA, Verdugo FJ: **Incontinentia pigmenti associated to cleft palate. Case report and literature review.** *Revista Odontológica Mexicana* 2012, **16**:58–61.
131. Jabbari A, Ralston J, Schaffer JV: **Incontinentia pigmenti.** *Dermatol Online J* 2010, **16**:9.
132. Jamnadas B, Agarwal R, Caddy CM: **A rare case of SCC in a young patient with incontinentia pigmenti.** *J Plast Reconstr Aesthet Surg* 2008, **61**:973–974. doi:10.1016/j.bjps.2007.09.050
133. Jandeck K, Kellner U, Foerester MH: **Succesful treatment of severe retinal vascular abnormalities in incontinentia pigmenti.** *Retina* 2004, **24**:631–633.
134. Jean-Baptiste S, O'Toole EA, Chen M, Guitart J, Paller A, Chan LS: **Expression of eotaxin, an eosinophil-selective chemokine, parallels eosinophil accumulation in the vesiculobullous stage of incontinentia pigmenti.** *Clin Exp Immunol* 2002, **127**:470–478.
135. Jessup CJ, Morgan SC, Cohen LM, Viders DE: **Incontinentia pigmenti: treatment of IP with topical tacrolimus.** *J Drugs Deramtol* 2009, **8**:944–946.
136. Jouet M, Stewart H, Landy S, Yates J, Yong SL, Harris A, Garret C, Hatchwell E, Read A, Donnai D, Kenwrick S: **Linkage analysis in 16 families with incontinentia pigmenti.** *Eur J Hum Genet* 1997, **5**:168–170.
137. Joy SP, Panda S, Kulkarni GB, Pal PK, Chickabasaviah YT, Battu RR: **Incontinentia pigmenti with sensorimotor polyneuropathy: A novel association.** *Neurol India* 2009, **57**: 813–815. doi:10.4103/0028-3886-59490
138. Kaczala GW, Messer MA, Poskitt KJ, Prendiville JS, Gardiner J, Senger C: **Therapy resistant neonatal seizures, linear vesicular rash, and unusually early neuroradiological changes: incontinentia pigmenti: a case report, literature review and insight into pathogenesis.** *Eur J Pediatr* 2008, **167**:979–983. doi:10.1007/s00431-007-0618-5
139. Kang SH, Kim S, Jung SH, Lee SG: **A case of incontinentia pigmenti with developmental brain malformation.** *J Korean Pediatr Soc* 2002, **45**:535–539.

140. Kasai T, Kato Z, Matsui E, Sakai A, Nishida T, Kondo N, Taga T: **Cerebral infarction in incontinentia pigmenti: the first report of a case evaluated by single photon emission computed tomography.** *Acta Paediatr* 1997, **86**:665–667.
141. Kaya TI, Tursen U, Ikizoglu G: **Therapeutic use of topical corticosteroids in the vesiculobullous lesions of incontinentia pigmenti.** *Clin Exp Dermatol* 2009, **34**:e611–613. doi:10.1111/j.1365-2230.2009.03301.x
142. Käsmann-Kellner B, Jurin-Bunte B, Ruprecht KW: **Incontinentia pigmenti (Bloch-Sulzberger-syndrome): case report and differential diagnosis to related dermato-ocular syndromes.** *Ophthalmologica* 1999, **213**:63–69.
143. Kenny D, Ramesh K, Murphy G, Keogan M, Gormally SM: **Somatic mosaicism for incontinentia pigmenti in a normal karyotype male infant.** *Ir J Med Sci* 2001, **170**(Suppl 3):58.
144. Kenwrick S, Woffendin H, Jakins T, Shuttleworth SG, Mayer E, Greenhalgh L, Whittaker J, Rugolotto S, Bardaro T, Esposito T, D'Urso M, Soli F, Turco A, Smahi A, Hamel-Teillac D, Lyonnet S, Bonnefont JP, Munnich A, Aradhya S, Kashork CD, Shaffer LG, Nelson DL, Levy M, Lewis RA; International IP Consortium: **Survival of male patients with incontinentia pigmenti carrying a lethal mutation can be explained by somatic mosaicism or Klinefelter syndrome.** *Am J Hum Genet* 2001, **69**:1210–1217. doi:10.1086/324591
145. Khan M, Pappert A: **Papulovesicular eruption in a newborn full-term female.** *J Am Acad Dermatol* 2009, **61**(Suppl 1):AB144.
146. Kikuchi I, Kaneko K, Hata M, Tatsuma N, Yamamoto M, Ibaragi N, Kawana S: **A case of incontinentia pigmenti and a review of extracutaneous complications in Japanese literature.** *J Pediatr Dermatol* 1999, **18**:161–164.
147. Kim BJ, Shin HS, Won CH, Lee JH, Kim KH, Kim MN, Ro BI, Kwon OS: **Incontinentia pigmenti: clinical observation of 40 Korean cases.** *J Korean Med Sci* 2006, **21**:474–477. doi:10.3346/jkms.2006.21.3.474
148. Kim MJ: **A genetic study in a patient with incontinentia pigmenti.** *Korean J Dermatol* 2011, **49**:164–166.
149. Kirchman TT, Levy ML, Lewis RA, Kanzler MH, Nelson DL, Scheuerle AE: **Gonadal mosaicism for incontinentia pigmenti in a healthy male.** *J Med Genet* 1995, **32**:887–890.
150. Kitakawa D, Fontes PC, Magalhães FA, Almeida JD, Cabral LA: **Incontinentia pigmenti presenting as hypodontia in a 3-year-old girl: a case report.** *J Med Case Reports* 2009, **3**:116. doi:10.1186/1752-1947-3-116
151. Kitamura M, Okamoto H, Uetsu N, Horio T**: A case of incontinentia pigmenti with possible recurrent inflammatory reaction in the third stage.** *Jpn J Clin Dermatol* 2005, **59**:1165–1167.
152. Kluk J, Loffeld A, McKeown C, Goodyear H: **Incontinentia pigmenti in a male infant with limited cutaneous expression.** *Arch Dis Child* 2010, **95**:A15. doi:10.1136/adc.2010.186338.35
153. Kmetz EC, Shashidhar Pai G, Burges GE: **Incontinentia pigmenti with a foreshortened hand: evidence for the significance of NFκB in human morphogenesis.** *Pediatr Dermatol* 2009, **26**:83–86. doi:10.1111/j.1525-1470.2008.00829.x
154. Konohana I, Tajima M: **Incontinentia pigmenti.** *Jpn J Clin Dermatol* 2003, **57**:6–7.
155. Kortüm AK, Büchau AS, Assmann B, Ruzicka T, Bruch-Gerharz D, Orth U, Kruse R: **Inflammatory stage of incontinentia pigmenti (Bloch-Sulzberger-Syndrom).** *Hautarzt* 2006, **57**:330–331. doi:10.1007/s00105-006-1116-9
156. Krämer N, Wolf NI, Harting I, Seitz A, Pöschl J, Ebinger F, Rating D: **Cortical and subcortical haemorrhagic necrosis as neonatal manifestation of incontinentia pigmenti.** Neuropediatrics 2004. doi:10.1055/s-2004-819367
157. Kutkowska-Kaźmierczak A, Obersztyn E, Rosińska-Borkowska D, Mazurczak T, Sobczyńska-Tomaszewska A, Mazurczak T: **Variable clinical expression of familial incontinentia pigmenti syndrome – presentation of three cases.** *Med Wieku Rozwoj* 2008, **12**:748–753.
158. Laguardia M, Lepore D, Pagliara MM, Baldascino A, De Santis R, D'Amico G, Angora C, Orazi L, Molle F, Balestrazzi E**: Fluorescein angiography in incontinentia pigmenti**. *Invest Ophthalmol Vis Sci* 2006, **47**:E-Abstract 5804.
159. Lai L, Wu J, Balaratnasingnam C: **Retinal sequelae of incontinentia pigmenti.** *Clin Experiment Ophthalmol* 2007, **35**:A90.
160. Lamounier FMC, Mansur CA, Corrêa GM, Mansur JS, Mansur JA: **Incontinentia pigmenti: two case reports.** *An Bras Dermatol* 2001, **76**:73–78.
161. Lee B, Reis M, Minimo C, Jacobson M: **Recurrence of the verrucous stage of the incontinentia pigmenti in an adult.** *J Cutan Pathol* 2010, **37**:190.
162. Lee JH, Im SA, Chun JS: **Serial changes in white matter lesions in a neonate with incontinentia pigmenti.** *Childs Nerv Syst* 2008, **24**:525–528. doi:10.1007/s00381-007-0550-z
163. Lee NC, Huang CH, Hwu WL, Chien YH, Chang YY, Chen CH, Ko TM: **Pseudogene-derived *IKBKG* gene mutations in incontinentia pigmenti.** *Clin Genet* 2009, **76**:417–419. doi:10.1111/j.1399-0004.2009.01232.x
164. Lee NK, Kim HK, Han YB: **A case of incontinentia pigmenti associated with ocular complications.** *J Korean Ophthalmol Soc* 1993, **34**:692–695.
165. Lee SY, Kim JH, Yu YS: **Bilateral fundus findings using examination under anesthesia in patients showing vitroretinopathy at unilateral posterior pole.** *J Korean Ophthalmol Soc* 2010, **51**:1099–1106.
166. Lee SY, Oh JS, Jung YT, Kim JS, Kim HS: **A case of incontinentia pigmenti with developmental delay.** *J Korean Child Neurol Soc* 2008, **16**:92–96.
167. Lee Y, Kim S, Kim K, Chang M: **Incontinentia pigmenti in a newborn with *NEMO* mutation.** *J Korean Med Sci* 2011, **26**:308–311. doi:10.3346/jkms.2011.26.2.308
168. Lehtmets A, Paluste M, Rattasep E, Zordania R, Kukk T: **Incontinentia pigmenti: a case report.** *Pediat Dermatol Suppl* 2001, **18**:88.

169. Leung AK, Kao CP, Robson WL: **Incontinentia pigmenti in an infant.** *J Pediatr* 2006, **149**:13.
170. Leyva-Sartori M, Cortez-Franco F, Carahyua-Perez D: **Incontinentia pigmenti. A case report.** *Dermatol Peru* 2006, **16**:70–73.
171. Li L, Song GW, Du JB, Liu JR, Xu FS, Liu XY, Zhang T: ***NEMO* delta 4-10 deletion of *NEMO* gene in Chinese incontinentia pigmenti cases.** *Zhonghua Er Ke Za Zhi* 2005, **43**:89–92.
172. Lin HK, Fu LS: **Concurrence of incontinentia pigmenti and Behçet's disease.** *J Chin Med Assoc* 2010, **73**:275–278.
173. Lin SY, Tsao PN, Hsieh WS, Hung CC, Su YN: **Genetic mutation in male patients with incontinentia pigmenti.** *J Formos Med Assoc* 2011, **110**:726. doi:10.1016/j.jfma.2011.09.012
174. Llano-Rivas I, Soler-Sánchez T, Málaga-Diéguez I, Fernández-Toral J: **Incontinentia pigmenti. Four patients with different clinical manifestations.** *An Pediatr (Barc)* 2012, **76**:156–160. doi:10.1016/j.anpedi.2011.09.008
175. Llombart B, Garcia L, Monteagudo C, Martín JM, Alonso V, Pinazo I, Calduch L, Jordá E: **Incontinentia pigmenti: a case with an unusual course.** *J Eur Acad Dermatol Venereol* 2005, **19**:394–396. doi:10.1111/j.1468-3083.2004.01141.x
176. Loh NR, Jadresic LP, Whitelaw A: **A genetic cause for neonatal encephalopathy: incontinentia pigmenti with *NEMO* mutation.** *Acta Paediatr* 2008, **97**:379–381. doi:10.1111/j.1651-2227.2007.00630.x
177. Lorda-Sánchez I, de Paula M, Bardaro T, Martín R, Villegas C, Ayuso C: **Incontinentia pigmenti male associated with with Klinefelter syndrome.** *An Esp Pediatr* 2001, **55**:177–178.
178. Lou H, Zhang L, Xiao W, Zhang J, Zhang M: **Nearly completely reversible brain abnormalities in a patient with incontinentia pigmenti.** *AJNR Am J Neuroradiol* 2008, **29**:431–433. doi:10.3174/ajnr.A0890
179. Löber R, Koehne P, Czernik C, Bührer C, Blume-Peytavi U: **Papulopustular lesions with blood eosinophilia in infants: manifestation of incontinentia pigmenti.** *Z Geburtshilfe Neonatol* 2009. doi:10.1055/s-0029-1223057
180. Lucarelli S, Lazzari S, Leonardi L, Frediani S, Federici T, Frediani T: **A neonatal case of rare blistering dermatitis.** *Arch Dis Child* 2008, **93**:ps303.
181. Macey-Dare LV, Goodman JR: **Incontinentia pigmenti: seven cases with dental manifestations.** *Int J Paediatr Dent* 1999, 9:293–297.
182. Maeda T, Suzuki J, Nakagawa T: **Familial incontinentia pigmenti with retinal vascular abnormality.** *Folia Ophthalmol Jpn* 1999, **50**:75–79.
183. Ma HY, Hsieh WS: **Disseminated skin manifestations in a neonate with incontinentia pigmenti.** *J Pediatr* 2011, **159**:510. doi:10.1016/j.jpeds.2011.04.015
184. Maingay-de Groof F, Lequin MH, Roofthooft DW, Oranje AP, de Coo IF, Bok LA, van der Spek PJ, Mancini GM, Govaert PP: **Extensive cerebral infarction in the newborn due to incontinentia pigmenti.** *Eur J Paediatr Neurol* 2008, **12**:284–289. doi:10.1016/j.ejpn.2007.09.001
185. Málaga Diéguez I, Bernardo Fernández B, Blanco Lago R, Llano Rivas I, Fernández Toral J: **Atypical onset of incontinentia pigmenti.** *An Pediatr (Barc)* 2009, **72**:1. doi:10.1016/j.anpedi.2009.09.016
186. Malvehy J, Palou J, Mascaró JM: **Painful subungual tumour in incontinentia pigmenti. Response to treatment with etretinate.** *Br J Dermatol* 1998, **138**:554–555.
187. Mane S: **Incontinentia pigmenti.** *Indian Pediatr* 2006, **43**:1103–1104.
188. Mangano S, Barbagallo A: **Incontinentia pigmenti: clinical and neuroradiologic features.** *Brain Dev* 1993, **15**:362–366. doi:10.1016/0387-7604(93)90122-O
189. Mansour S, Woffendin H, Mitton S, Jeffery I, Jakins T, Kenwrick S, Murday VA: **Incontinentia pigmenti in a surviving male is accompanied by hypohidrotic ectodermal dysplasia and recurrent infection.** *Am J Med Genet* 2001, **99**:172–177.
190. Marchionatti C, Carrasco F, Pardo Argerich M, Di Servi O: **A case study of neonatal pigmenti incontinence.** *In: Abstract book, 7. Jornada Nacional de Pediatras en Formación Ciudad de Buenos Aires, Argentina.* 2007:17.
191. Marinoaica S, Predoi L, Dragan D: **Incontinentia pigmenti: about one case.** *J Eur Acad Dermatol Venereol* 2003, **17**:283.
192. Márquez Balbás G, González-Enseñat MA, Vicente A, Creus-Vila L, Antón J, Umbert-Millet P: **Incontinentia pigmenti and bipolar aphthosis: an unusual combination.** *ISRN Dermatol* 2011, **2011**:814186. doi:10.5402/2011/814186
193. Martinez-Pomar N, Munoz-Saa I, Heine-Suner D, Martin A, Smahi A, Matamoros N: **A new mutation in exon 7 of *NEMO* gene: late skewed X-chromosome inactivation in an incontinentia pigmenti female patient with immunodeficiency.** *Hum Genet* 2005, **118**:458–465. doi:10.1007/s00439-005-0068-y
194. Matelzonas T, Ruvertoni M, Reyno S, Pinchak MC: **Incontinentia pigmenti. Neonatal presentation. Apropos of a case.** *Arch Pediatr Urug* 2010, **81**:23–29.
195. Matsumoto N, Takahashi S, Toriumi N, Sarashina T, Makita Y, Tachibana Y, Fujieda K: **Acute disseminated encephalomyelitis in an infant with incontinentia pigmenti.** *Brain Dev* 2009, **31**:625–628. doi:10.1016/j.braindev.2008.08.010
196. Mayer EJ, Shuttleworth GN, Greenhalgh KL, Sansom JE, Grey RH, Kenwrick S: **Novel corneal features in two males with incontinentia pigmenti.** *Br J Ophthalmol* 2003, **87**:554–556. doi:10.1136/bjo.87.5.554
197. McBrien J, Irvine AD, Phelan E, Brosnahan D, Webb D: **Incontinentia pigmenti presenting with neonatal seizures and ischaemic brain injury.** *Br J Dermatol* 2004, **151**:261. doi:10.1111/j.1365-2133.2004.06034.x

198. McNiff JM: **Subungal tumors of incontinentia pigmenti.** In: *ASDP 43rd Annual Meeting, Evening Slide Symposium, Chicago, Illinois.* 2006:18.
199. Meallet M, Song J, Stout JT: **An extreme case of retinal avascularity in a female neonate with incontinentia pigmenti.** *Retina* 2004, **24**:613–615.
200. Minić S, Novotny GEK, Trpinac D, Obradović M: **Clinical features of incontinentia pigmenti with emphasis on oral and dental abnormalities.** *Clin Oral Invest* 2006, **10**:343–347. doi:10.1007/s00784-006-0066-z
201. Minić S, Trpinac D, Obradović M, Novotny GEK, Gabriel HD, Kuhn M: **Incontinentia pigmenti with ultrastructuraly disordered leukocytes.** *J Clin Pathol* 2010, **63**:657–659. doi:10.1136/jcp.2009.074203
202. Miteva L, Nikolova A: **Incontinentia pigmenti: a case associated with cardiovascular anomalies.** *Pediatr Dermatol* 2001, **18**:54–56.
203. Montes CM, Maize JC, Guerry-Force ML: **Incontinentia pigmenti with painful subungual tumors: a two-generation study.** *J Am Acad Dermatol* 2004, **50**:S45–52. doi:10.1016/S0190-9622(03)02467-8
204. Moore K, Lam JM: **The toddler with 1 striped leg: a linear papular rash.** *CMAJ* 2009, **180**:947–948. doi:10.1503/cmaj.082086
205. Motamedi MH, Lotfi A, Azizi T, Moshref M, Farhadi S: **Incontinentia pigmenti.** *Indian J Pathol Microbiol* 2010, **53**:302–304.
206. Mühlenstädt E, Eigelshoven S, Hoff NP, Reifenberger J, Homey B, Bruch-Gerharz D: **Incontinentia pigmenti (Bloch-Sulzberger syndrome).** *Hautarzt* 2010, **61**:831–833. doi:10.1007/s00105-010-2046-0
207. Nagase T, Takanashi M, Takada H, Ohmori K: **Extensive vesiculobullous eruption following limited ruby laser treatment for incontinentia pigmenti: a case report.** *Australas J Dermatol* 1997, **38**:155–157.
208. Narayan S, De Berker D, Oxley J: **Subungual keratoacanthoma of incontinentia pigmenti.** *Br J Dermatol* 2001, **145**(Suppl. 59):114.
209. Nettesheim S, Nöh F, Skopnik F: **Incontinentia pigmenti in a differential diagnosis of vesicular skin diseases newborns.** *Z Geburtshilfe Neonatol* 2007. doi:10.1055/s-2007-983189
210. Neumeister-Chisholm E, Thomas K, O'Neal B, Williams J, Proud V: **Redefining our understanding of incontinentia pigmenti: literature review and report of a new case of IP in a healthy male infant.** *J Genet Counsel* 2010, **19**:711.
211. Nguyen JK, Brady-McCreery KM: **Laser photocoagulation in preproliferative retinopathy of incontinentia pigmenti.** *J AAPOS* 2001, **5**:258–259. doi:10.1067/mpa.2001.117098
212. Nicolaou N, Graham-Brown RA: **Nail dystrophy, an unusual presentation of incontinentia pigmenti.** *Br J Dermatol* 2003, **149**:1286–1288.
213. Nogueira A, Lisboa C, Eloy C, Mota A, Azevedo F: **Vesicular rash in a newborn. Incontinentia pigmenti.** *Indian J Dermatol Venereol Leprol* 2009, **75**:330. doi:10.4103/0378-6323.51255
214. Nouri-Merchaoui S, Mahdhaoui N, Methlouthi J, Zakhama R, Seboui H: **Neonatal seizures revealing incontinentia pigmenti.** *Arch Pediatr* 2011, **18**:1095–1099. doi:10.1016/j.arcped.2011.05.023
215. Nso Roca AP, Baquero-Artigao F, García-Miguel MJ, Guerrero Vázquez J, Guerrero Fernández J, Vicente Cuevas y R. de Lucas Laguna M: **Incontinentia pigmenti. Initial and long-term characteristics.** *An Pediatr (Barc)* 2008, **68**: 9–12.
216. Obermann M, Weber R: **Concomitant diagnosis of sarcoidosis and incontinentia pigmenti in an epileptic patient.** *Eur J Neurol* 2008, **15**:e36–37. doi:10.1111/j.1468-1331.2008.02095.x
217. Odent S, Le Marec B, Smahi A, Hors-Cayla C, Milon J, Jouan H, Laurent MC, Borg AM: **The spontaneous termination of pregnancies of male fetuses achieved in incontinentia pigmenti (apropos of a family).** *J Gynecol Obstet Biol Reprod (Paris)* 1997, **26**:633–636.
218. O'Doherty M, Mc Creery K, Green AJ, Tuwir I, Brosnahan D: **Incontinentia pigmenti – ophthalmological observation of a series of cases and review of the literature.** *Br J Ophthalmol* 2011, **95**:11–16. doi:10.1136/bjo.2009.164434
219. Ofuji S, Okada M, Kazama S, Yamamoto O: **Incontinentia pigmenti in a boy: a helpful diagnostic value of smear cytology.** *Rinsho Derma (Tokyo)* 2006, **48**:741–743.
220. Ohira A, Azuma N: **Vitrectomy of proliferative retinopathy in two patients with incontinentia pigmenti (Bloch-Sulzberger syndrome).** *Ophthalmology* 2003, **45**:125–130.
221. Okan F, Yapici Z, Bulbul A: **Incontinentia pigmenti mimicking a herpes simplex virus infection in the newborn.** *Childs Nerv Syst* 2008, **24**:149–151. doi:10.1007/s00381-007-0406-6
222. Okita M, Nakanishi G, Fujimoto N, Kishida M, Tanaka T: **Incontinentia pigmenti with *NEMO* mutation in a Japanese family.** J Dermatol 2012. doi:10.1111/j.1346-8138.2011.01496.x.
223. Osório F, Magina S, Nogueira A, Azevedo F: **Incontinentia Pigmenti with vesicular stage in utero.** *Dermatol Online J* 2010, **16**:13.
224. Oswal HN, Konia T, Rodriguez R: **Incontinentia pigmenti.** *J Cutan Pathol* 2006, **33**:103.
225. Oyama R, Yoshimura E, Ichise K, Yano T: **Anesthetic management of a patient with Bloch-Sulzberger syndrome/incontinentia pigmenti.** *J Clin Anesth* 2005, **29**:1071–1072.
226. Pacheco TR, Levy M, Collyer JC, de Parra NP, Parra CA, Garay M, Aprea G, Moreno S, Mancini AJ, Paller AS: **Incontinentia pigmenti in male patients.** *J Am Acad Dermatol* 2006, **55:**251–255. doi:10.1016/j.jaad.2005.12.015
227. Parrish JE, Scheuerle AE, Lewis RA, Levy ML, Nelson DL: **Selection against mutant alleles in blood leukocytes is a consistent feature in incontinentia pigmenti type 2.** *Hum Mol Genet* 1996, **5**:1777–1783. doi:10.1093/hmg/5.11.1777

228. Pascual-Castroviejo I, Pascual-Pascual SI, Velázquez-Fragua R, Martinez V: **Incontinentia pigmenti: clinical and neuroimaging findings in a series of 12 patients.** *Neurologia* 2006, **21**:239–248.
229. Patrizi A, Neri I, Guareschi E, Cocchi G: **Bullous recurrent eruption of incontinentia pigmenti.** *Pediatr Dermatol* 2004, **21**:613–614.
230. Pauly E, Linderkamp O, Pöschl J: **Incontinentia pigmenti in combination with decreased IgG subclass concentrations in a female newborn.** *Biol Neonate* 2005, **88**:172–174.
231. Pearlman J, Griego RD, Levy ML, Friedman J: **An unusual presentation of incontinentia pigmenti in a 4-month-old girl.** *Pediatr Dermatol* 1996, **13**:47–50.
232. Pellegrino RJ, Shah AJ: **Vascular occlusion associated with incontinentia pigmenti.** *Pediatr Neurol* 1994, **10**:73–74.
233. Pereira MA, Mesquita LA, Budel AR, Cabral CS, Feltrim AS: **X-linked incontinentia pigmenti or Bloch-Sulzberger syndrome: a case report.** *An Bras Dermatol* 2010, **85**:372–375.
234. Pettigrew R, Kuo HC, Scriven P, Rowell P, Pal K, Handyside A, Braude P, Ogilvie CM: **A pregnancy following PGD for X-linked dominant incontinentia pigmenti (Bloch-Sulzberger syndrome): case report.** *Hum Reprod* 2000, **15**:2650–2652.
235. Pfau A, Landthaler M: **Recurrent inflammation in incontinentia pigmenti of a seven-year-old child.** *Dermatology* 1995, **191**:161–163.
236. Phan TA, Wargon O, Turner AM: **Incontinentia pigmenti case series: clinical spectrum of incontinentia pigmenti in 53 female patients and their relatives.** *Clin Exp Dermatol* 2005, **30**:474–480. doi:10.1111/j.1365-2230.2005.01848.x
237. Piccoli GB, Attini R, Vigotti FN, C Naretto C , Fassio F, Randone O, Restagno G, Todros T, Roccatello D: **Nemo syndrome (incontinentia pigmenti) and systemic lupus erythematosus: a new disease association.** *Lupus* 2012, **21**:675–681. doi:10.1177/0961203311433140
238. Pock L, Drlík L: **Incontinentia pigmenti posterior – macular stage.** *Čs Derm* 1999, **74**:253–255.
239. Portaleone D, Taroni E, Micheli S, Moioli M, Pedrazzini A, Carnelli V: **Incontinentia pigmenti: a case report.** *Pediatr Med Chir* 2007, **29**:343–345.
240. Pörksen G, Pfeiffer C, Hahn G, Poppe M, Friebel D, Kreuz F, Gahr M: **Neonatal seizures in two sisters with incontinentia pigmenti.** *Neuropediatrics* 2004, **35**:139–142. doi:10.1055/s-2004-815837
241. Purohit S, Singhi MK, Khullar R, Kalla G: **Incontinentia pigmenti.** *Indian J Dermatol Venereol Leprol* 1995, **61**:295–296.
242. Ranchod TM, Trese MT: **Regression of retinal neovascularization after laser photocoagulation in incontinentia pigmenti.** *Retina* 2010, **30**:708–709.
243. Regueras Santos L, Morales Sánchez R, Castañón López L, Mata Zubillaga D, Martínez Castellano F: **Early neurological symptoms in patients with incontinentia pigmenti.** *Anales de Pediatría* 2009, **71**:576–578. doi:10.1016/j.anpedi.2009.07.032
244. Reutter J, Goldstein J, Prose N, McDonald M, Selim M: **Incontinentia pigmenti in male with mosaic Klinefelter syndrome: the role of FISH analysis.** *J Cutan Pathol* 2005, **32**:112.
245. Rhee K, Kim YS, Kim IW, Huh K: **A case of progressive proliferative retinopathy in incontinentia pigmenti after photocoagulation.** *J Korean Ophthalmol Soc* 2000, **41**:272–275.
246. Roberts JL, Morrow B, Vega-Rich C, Salafia CM, Nitowsky HM: **Incontinentia pigmenti in a newborn male infant with DNA confirmation.** *Am J Med Genet* 1998, **75**:159–163.
247. Rodrigues V, Diamantino F, Voutsen O,Cunha MS, Barroso R, Lopes MJP, Carreiro H: **Incontinentia pigmenti in the neonatal period.** *BMJ Case Reports* 2011. doi:10.1136/bcr.01.2011.3708
248. Rola M, Martins T, Melo MJ, Gomes R, Roseira J, Souto A: **Incontinence of pigment.** *An Pediatr (Barc)* 2004, **60**:601–602.
249. Rosman F, Cerqueira AMM, Rosman A, Mourao CN: **Pigmentary incontinence, type 2, male newborn surviving 29 days: Case report.** *J Am Acad Dermatol* 2011, **64**(Suppl 1):AB87.
250. Sáez-ce-Ocariz M, Orozco-Covarrubias L, Durán-McKinster C, Palacios-López C, Ruíz-Maldonaldo: **Incontinentia pigmenti: estado actual y experiencia en el Instituto Nacional de Pediatría de México.** *Dermatol Pediatr Lat* 2006, **4**:101–110.
251. Sahn EE, Davidson LS: **Incontinentia pigmenti: three cases with unusual features.** *J Am Acad Dermatol* 1994, **31**:852–857.
252. Sakai H, Minami M, Satoh E, Matsuo S, Iizuka H: **Keratoacanthoma developing on a pigmented patch in incontinentia pigmenti.** *Dermatology* 2000, **200**:258–261. doi:10.1159/000018371
253. Sanghi G, Dogra MR, Ray M, Gupta A: **Predominant exudative retinopathy in incontinentia pigmenti and clinical course after peripheral laser photocoagulation.** *Indian J Ophthalmol* 2011, **59**:255–256. doi:10.4103/0301-4738.81022
254. Sanka R, Kumar M: **An unusual newborn rash.** *Fetal Pediatr Pathol* 2004, **23**:275–279. doi:10.1080/15227950490923750
255. Sashikumar P, Mukherjee S: **Neonatal incontinentia pigmenti.** *BMJ Case Reports* 2010. doi:10.1136/bcr.04.2010.2939
256. Sato H, Seto H, Taninaka Y: **Identical twins of incontinentia pigmenti: a case report.** *Skin Research* 2004, **3**:198–201.

257. Scardamaglia L, Howard A, Sinclair R: **Twenty-nail dystrophy in a girl with incontinentia pigmenti.** *Australas J Dermatol* 2003, **44**:71–73.
258. Schaller J, Schaller S: **A rare variant of incontinentia pigmenti – disseminated papules and vesicles.** *J Eur Acad Dermatol Venereol* 1996, **6**:47–49.
259. Scheuerle AE: **Male cases of incontinentia pigmenti: case report and review.** *Am J Med Genet* 1998, **77**:201–218.
260. Scheuerle A, Lewis RA, Levy ML, Nelson DL: **De novo mutation in three families with multigenerational incontinentia pigmenti.** *Am J Hum Genet* 1994, **55**:1279–1281.
261. Schmeling H, Wohlrab J, Mathony K, Gaber G, Lieser U, Burdach S, Horneff G: **Incontinentia pigmenti Bloch-Sulzberger.** *Monatsschr Kinderheilkd* 2001, **149**:41–44.
262. Sebban-Benin H, Pescatore A, Fusco F, Pascuale V, Gautheron J, Yamaoka S, Moncla A, Ursini MV, Courtois G: **Identification of TRAF6-dependent *NEMO* polyubiquitination sites through analysis of a new *NEMO* mutation causing incontinentia pigmenti.** *Hum Mol Genet* 2007, **16**:2805–2815. doi:10.1093/hmg/ddm237
263. Seeborg FO, Rosenblatt HM, Shearer WT, Norski LM, Pacheco SE: **Incontinentia pigmenti associated with a severe form of immune deficiency.** *J Allergy Clin Immunol* 2002, **109**:S187.
264. Selvadurai D, Salomão DR, Baratz KH: **Corneal abnormalities in incontinentia pigmenti: histopathological and confocal correlations.** *Cornea* 2008, **27**:833–836.
265. Senturk N, Aydin F, Haciomeroglu P, Yildiz L, Totan M, Canturk T, Turanli AY: **Pulmonary tuberculosis and cutaneous mycobacterial infection in a patient with incontinentia pigmenti.** *Pediatr Dermatol* 2004, **2**:660–663.
266. Seol KH, Lee GH, Kim GH, Lee HS, Lee JD: **Incontinentia pigmenti in a mother and her daughter.** *J Korean Soc Neonatal* 2001, **8**:276–280.
267. Shah L, Balakumar G: **Visual diagnosis: a persistent newborn rash.** *Pediatr Rev* 2007, **28**:429–432.
268. Shah SN, Gibbs S, Upton CJ, Pickworth FE, Garioch JJ: **Incontinentia pigmenti associated with cerebral palsy and cerebral leukomalacia: a case report and literature review**. *Pediatr Dermatol* 2003, **20**:491–494.
269. Shaikh S, Trese M, Archer SM: **Fluorescein angiographic findings in incontinentia pigmenti.** *Retina* 2004, **24**:628–629.
270. Shatkin BT, Hunter JG, Song IC: **Familial subungual keratoacanthoma in association with ectodermal dysplasia.** *Plast Reconstr Surg* 1993, **92**:528–531.
271. Shields CL, Eagle RC Jr, Shah RM, Tabassian A, Shields JA: **Multifocal hypopigmented retinal pigment epithelial lesions in incontinentia pigmenti.** *Retina* 2006, **26**:328–333.
272. Silan F, Aydogan I, Kavak A, Bardaro T, D'Urso M: **Incontinentia pigmenti with *NEMO* mutation in a Turkish family.** *Int J Dermatol* 2004, **43**:527–529.
273. Smahi A, Hyden-Granskog C, Peterlin B, Vabres P, Heuertz S, Fulchignoni-Lataud MC, Dahl N, Labrune P, Le Marec B, Piussan C, Taleb A, Koskull H von, Hors-Cayla MC: **The gene for the familial form of incontinentia pigmenti (IP2) maps to the distal part of Xq28.** *Hum Mol Genet* 1994, **3**:273–278. doi:10.1038/35013114
274. Solovastru L, Taranu T, Amalinei C, Taranu T: **Particularities of clinic expression in a case of Bloch-Sulzberger syndrome.** *J Eur Acad Dermatol Venereol* 2005, **19**:776–777. doi:10.1111/j.1468-3083.2005.01269.x
275. Soltau JB, Lueder GT: **Bilateral macular lesions in incontinentia pigmenti. Bloch-Sulzberger syndrome.** *Retina* 1996, **16**:38–41.
276. Song MJ, Chae JH, Park EA, Ki CS: **The common NF-κB essential modulator (*NEMO*) gene rearrangement in Korean patients with incontinentia pigmenti.** *J Korean Med Sci* 2010, **25**:1513–1517. doi:10.3346/jkms.2010.25.10.1513
277. Sotiriou E, Patsatsi A, Sotiriadis D, Papagaryfallou I, Chrysomallis F: **Incontinentia pigmenti: a genodermatosis with a significant phenotypic variety – a three-case report in family.** *J Eur Acad Dermatol Venereol* 2003, **17**:279.
278. Stitt WZ, Scott GA, Caserta M, Goldsmith LA: **Coexistence of incontinentia pigmenti and neonatal herpes simplex virus infection.** *Pediatr Dermatol* 1998, **15**:112–115.
279. Su PH, Chen JY, Yu JS, Su CM, Huang TC, Chen SJ: **De novo incontinentia pigmenti in female twins.** *Acta Paediatr Taiwan* 2004, **45**:178–180.
280. Succi IB, Rosman FC, Oliveira EF: **Do you know this syndrome?** *An Bras Dermatol* 2011, **86**:608–610.
281. Syriopoulou T, Fourlani E, Polykarpou E, Michail E: **Incontinentia pigmenti** – **Case report.** In: *Abstract UENPS.60,* 2008:530. doi:10.1016/j.earlhumdev.2008.09.076
282. Tada H, Yoshida S, Yamaji Y, Fujisawa K, Ishibashi T: ***NEMO* mutational analysis in a Japanese family with incontinentia pigmenti.** *Eye* 2007, **21**:888–890. doi:10.1038/sj.eye.6702770
283. Takizawa M, Oba C, Kidokoro H, Nagasawa M, Kaneda T, Kimoto H: **A case of incontinentia pigmenti with clonic hemiconvulsion in infancy.** *J Saitama Children's Medical Center* 2004, **21**:31–35.
284. Tanboga I, Kargul B, Ergeneli S, Aydin MY, Atasu M: **Clinical features of incontinentia pigmenti with emphasis on dermatoglyphic findings.** *J Clin Pediatr Dent* 2001, **25**:161–165.
285. Tekin N, Uçar B, Saraçoglu ZN, Koçak AK, Urer S, Yakut A: **Diagnosis and follow up in four cases of incontinentia pigmenti.** *Pediatr Int* 2000, **42**:557–560.
286. Thakur S, Puri RD, Kohli S, Saxena R, Verma IC: **Utility of molecular studies in incontinentia pigmenti patients.** *Indian J Med Res* 2011, **133**:442–445.
287. Tnacheri Ouazzani B, Guedira K, Dali H, Laghmari M, Ibrahimy W, Daoudi R, Sefiani A, Chakir M, Jiddane M, Mohcine Z: **Incontinentia pigmenti: a case study.** *J Fr Ophtalmol* 2007, **30**:844.

288. Tomaraei SN, Bajwa RP, Dhiman P, Marwaha RK: **Incontinentia pigmenti (Bloch-Sulzberger syndrome): report of a case and review of the Indian literature.** *Indian J Pediatr* 1995, **62**:118–122.
289. Topham EJ, Simpson Dent SL, Child FJ: **Case 1. Incontinentia pigmenti.** *Clin Exp Dermatol* 2003, **28**: 103–104.
290. Türkmen M, Eliaçik K, Temoçin K, Savk E, Tosun A, Dikicioğlu E: **A rare cause of neonatal seizure: incontinentia pigmenti.** *Turk J Pediatr* 2007, **49**:327–330.
291. Urban J, Toruniowa B, Janniger CK, Czelej D, Schwartz RA: **Incontinentia pigmenti (Bloch-Sulzberger syndrome): multisystem disease observed in two generations.** *Cutis* 1996, **58**:329–336.
292. Valentí C, López A, Laplaza Y, Aseguinolaza B, Olaizola Y: **Incontinentia pigmenti: a case report.** *Vichows Arch* 2001, **439**:375.
293. Van den Steen E, Bottenberg P, Bonduelle M: **Dental anomalies associated with incontinentia pigmenti or Bloch-Sulzberger syndrome.** *Rev Belge Med Dent* 1984 2004, **59**:94–99.
294. Van Leeuwen RL, Wintzen M, Van Praag MCG: **Incontinentia pigmenti: an extensive second episode of a "first-stage" vesicobullous eruption.** *Pediatr Dermatol* 2000, **17**:70.
295. Vehring KH, Kurlemann G, Traupe H, Bonsmann G, Gerding H, Möllmann S, Hamm H: **Incontinentia pigmenti bei einem männlichen Säugling.** *Hautarzt* 1993, **44**:726–730.
296. Vicente-Villa A, Lamas JV, Pascual AM, Cuesta DL, Marfa MP, González-Enseñat MA: **Incontinentia pigmenti: a report of ten cases.** *Eur J Pediatr* 2001, **160**:64–65.
297. Wald KJ, Mehta MC, Katsumi O, Sabates NR, Hirose T: **Retinal detachments in incontinentia pigmenti.** *Arch Ophthalmol* 1993, **111**:614–617.
298. Wammanda RD, Idris HW, Musa S, Chom ND, Akuyam SA: **Pigmenti associated with precocious puberty: case report.** *Ann Afr Med* 2006, **5**:111–113.
299. Warren RB, Parslew R: **A linear blistering in a monozygotic twin.** *J Eur Acad Dermatol Venereol* 2004, **18**:318.
300. Watanabe S, Morita A: **A case of incontinentia pigmenti in a male infant.** *Nishinihon J Dermatol* 2008, **70**:406–409.
301. Wiederholt T, Poblete-Gutiérrez P, Ott H, Lehmann S, Grussendorf-Conen EI, Beermann T, Frank J: **Incontinentia pigmenti in a five-week-old girl.** *Hautarzt* 2004, **55**:999–1001. doi:10.1007/s00105-004-0792-6
302. Winterberg DH, van Tijn DA, Smitt JH, Winterberg S, Vomberg PP: **Two neonates with vesicular skin lesions due to incontinentia pigmenti.** *Ned Tijdschr Geneeskd* 2001, **145**:2178–2182.
303. Woffendin H, Jakins T, Jouet M, Stewart H, Landy S, Haan E, Harris A, Donnai D, Read A, Kenwrick S: **X-inactivation and marker studies in three families with incontinentia pigmenti: implications for counselling and gene localisation.** *Clin Genet* 1999, **55**:55–60.
304. Wolf NI, Krämer N, Harting I, Seitz A, Ebinger F, Pöschl J, Rating D: **Diffuse cortical necrosis in a neonate with incontinentia pigmenti and an encephalitis-like presentation.** *AJNR Am J Neuroradiol* 2005, **26**:1580–1582.
305. Wong GAE, Willoughby CE, Parslew R, Kaye SB: **Importance of screening for sight-threatening retinopathy in incontinentia pigmenti.** *Br J Dermatol* 2002, **147**:412.
306. Woo MJ, Jung BC, Kim SW, Jun JB: **A case of incontinentia pigmenti associated with right cerebral hemiatrophy.** *Korean J Dermatol* 2002, **40**:574–576.
307. Wong GAE, Willoughby CE, Parslew R, Kaye SB: **The importance of screening for sight-threatening retinopathy in incontinentia pigmenti.** *Pediatr Dermatol* 2004, **21**:242–245.
308. Wu HP, Wang YL, Chang HH, Huang GF, Guo MK: **Dental anomalies in two patients with incontinentia pigmenti.** *J Formos Med Assoc* 2005, **104**:427–430.
309. Yamashiro T, Nakagawa K, Takada K: **Case report: orthodontic treatment of dental problems in incontinentia pigmenti.** *Angle Orthod* 1998, **68**:281–284.
310. Yang JH, Ma SY, Tsai CH: **Destructive encephalopathy in incontinentia pigmenti: a case report.** *J Dermatol* 1995, **22**:340–343.
311. Yasuda K, Minami N, Yoshikawa Y, Fukuda S, Yamaguchi S: **Severe pulmonary hypertension in an infant girl with incontinentia pigmenti.** *Cardiol Young* 2010, **20**(Suppl 1):326.
312. Yasuda M, Amano H, Tamura A, Ishikawa O, Harigaya A: **A case of incontinentia pigmenti with retinodialysis and seizure.** *Jpn J Clin Dermatol* 2003, **57**:265–268.
313. Yoshida M, Oiso N, Kimura M, Itoh T, Kawada A: **Skin ulcer mimicking pyoderma gangrenosum in a patient with incontinentia pigmenti.** *J Dermatol* 2011, **38**:1019–1021. doi:10.1111/j.1346-8138.2010.01160.x.
314. Yoshikawa H, Uehara Y, Abe T, Oda Y: **Disappearance of a white matter lesion in incontinentia pigmenti.** *Pediatr Neurol* 2000, **23**:364–367.
315. Yoshizawa S, Hatcho M: **Unilateral incontinentia pigmenti.** *Rinsho Derma (Tokyo)* 2006, **48**:745–758.
316. Young A, Manolson P, Cohen B, Klapper M, Barrett T: **Painful subungal dyskeratotic tumors in incontinentia pigmenti.** *J Am Acad Dermatol* 2005, **52**:726–729. doi:10.1016/j.jaad.2004.11.056
317. Yu YS, Park KC: **Retinal vascular changes and treatment of incontinentia pigmenti eyes.** *J Korean Ophthalmol Soc* 1998, **39**:213–220.
318. Zou CC, Zhao ZY: **Clinical and molecular analysis of NF-κB essential modulator in Chinese incontinentia pigmenti patients.** *Int J Dermatol* 2007, **46**:1017–1022.
